# Iron-Catalyzed Cross-Coupling of α-Allenyl Esters with Grignard Reagents for the Synthesis of 1,3-Dienes

**DOI:** 10.1021/acs.orglett.2c03916

**Published:** 2023-01-04

**Authors:** Wei-Jun Kong, Simon N. Kessler, Haibo Wu, Jan-E. Bäckvall

**Affiliations:** Department of Organic Chemistry, Arrhenius Laboratory, Stockholm University, 10691 Stockholm, Sweden

## Abstract

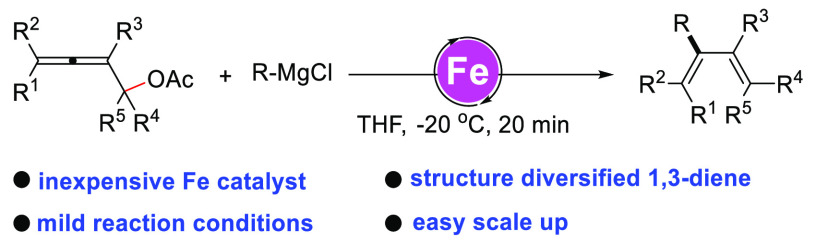

Structurally diverse 1,3-dienes are valuable building blocks in organic synthesis. Herein we report the iron-catalyzed coupling between α-allenyl esters and Grignard reagents, which provides a fast and practical approach to a variety of complex substituted 1,3-dienes. The reaction involves an inexpensive iron catalyst, mild reaction conditions, and provides easy scale up.

1,3-Dienes are important structural motifs in natural products and valuable building blocks in organic synthesis.^1^ A plethora of transformations involving 1,3-dienes have been developed including Diels–Alder addition,^2^ hydrofunctionalization,^3^ and difunctionalization.^4^ Traditionally, olefination reactions such as Wittig, Horner–Wadsworth–Emmons, and Julia–Kocienski reactions are applied for the synthesis of 1,3-dienes.^5^ However, these methods sometimes suffer from the drawbacks of low atom economy, poor functional group tolerance, or unsatisfactory stereoselectivity. Methodologies providing simple and efficient access to structurally diverse substituted 1,3-dienes are still in high demand.

In recent years, transition metal catalysis has become a powerful tool for the synthesis of 1,3-dienes through reactions including Mizoroki–Heck reactions, cross-coupling, ene-yne metathesis, isomerization, and so on (Scheme 1A).^6^ The control of regio- and stereoselectivity still constitutes the main challenge in these transition metal-catalyzed 1,3-diene synthesis reactions. While palladium catalysts play a major role in these reactions, methods based on inexpensive 3d metal catalysts, such as iron,^7^ copper,^8^ and nickel,^9^ are still underdeveloped. Iron catalysis has received considerable attention in organic chemistry due to its high earth abundance and low toxicity. Notable reactions catalyzed by iron complexes include cross couplings,^10^ oxidations,^11^ and C–H functionalizations,^12^ among others.

**Scheme 1:**
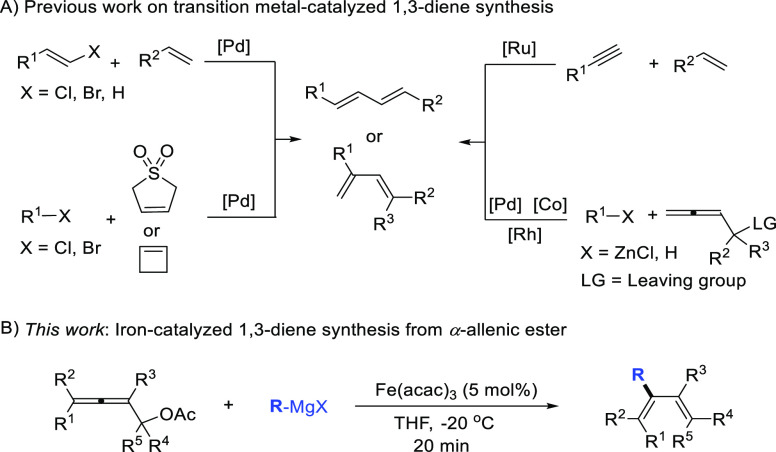
Previous Work on Transition Metal-Catalyzed 1,3-Diene Synthesis (A) and This Work (B)

α-Allenyl esters or carbonates have been explored in palladium- and iridium-catalyzed asymmetric nucleophilic substitution (S_N_) reaction for chiral allene synthesis.^13^ Furthermore, they were also found to be valuable reagents for 1,3-diene synthesis as demonstrated in palladium-, rhodium-, and cobalt-catalyzed coupling reactions (Scheme 1A, lower part to the right).^14^ However, most of these reactions are limited to sterically unhindered α-allenyl derivatives, such as terminal allenes. In previous studies, our group has demonstrated the versatility of iron catalysis in allene synthesis with propargylic esters and ethers as substrates via an S_N_2′ pathway.^15^ Therefore, it is conceivable that 1,3-dienes could be accessed from α-allenyl derivatives through an iron-catalyzed reaction. Herein, we report a mild and efficient approach for the regio- and stereoselective synthesis of 1,3-dienes from structurally diverse α-allenyl esters using iron catalysis (Scheme 1B).

We initiated the envisaged iron-catalyzed reaction with α-allenic acetates **1a** (R = OAc) and benzyl magnesium chloride **2a** as starting materials. The desired 1,3-diene **3aa** was obtained in 93% NMR yield with an *E*/*Z* ratio of 7.5:1 using 5.0 mol % of tris(acetylacetonate)iron (Fe(acac)_3_) as precatalyst and diethyl ether (Et_2_O) as solvent at −20 °C for 20 min (Table 1, entry 1). On isolation with column chromatography on silica (92% yield) the *E*/*Z* ratio decreased to 5.0:1. When ferric chloride (FeCl_3_) was used as catalyst, a similar result was obtained with a slight decrease in yield (85%, Table 1, entry 2). However, ferrous chloride (FeCl_2_) only afforded **3aa** in 16% yield (entry 3). Solvents such as toluene or tetrahydrofuran (THF) also delivered **3aa** in excellent yields, but the stereoselectivity was unsatisfactory with *E*/*Z* ratios of 3.9:1 and 2.5:1, respectively (entry 4 and 5). When methoxide was used as leaving group, the reaction delivered **3aa** in only 36% yield with an *E*/*Z* ratio of 17:1 (entry 6). The use of pivalate **1a** (R = Piv) afforded the diene product in 85% yield with an *E*/*Z* ratio of 4.7:1. The reaction of acetate **1a** (R = Ac) at elevated temperature (0 °C) gave a similar result (92% yield and *E*/*Z* = 7.5:1) as that at −20 °C (entry 8 vs entry 1, Table 1). The addition of catalytic amounts of tetramethylethylenediamine (TMEDA) improved the yield to 96%, but the *E*/*Z* ratio decreased to 3.5:1 (entry 9, Table 1). A control experiment proved the indispensability of the iron catalyst in this reaction (entry 10, Table 1). To rule out the possibility that the reactivity was due to trace amounts of impurities such as palladium or copper in the iron precatalyst, we ran the reaction with the amounts of Pd(OAc)_2_ or CuI that would correspond to 0.1 wt % of the Fe(acac)_3_ (5 mol %) used. In both cases <5% of product **3aa** was formed (entry 11, Table 1).

**Table 1:**
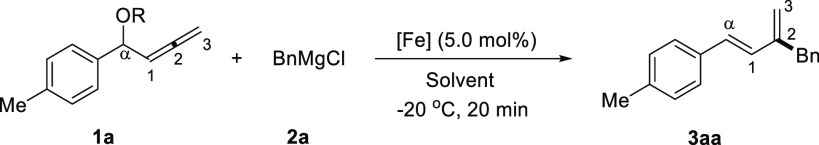
Optimization of Iron-Catalyzed 1,3-Diene Synthesis from α-Allenyl Derivatives^a^

entry	R	[Fe]	solvent	**3aa** (%)	*E*/*Z*
**1**	**Ac**	**Fe(acac)**_**3**_	**Et**_**2**_**O**	**93(92)**^b^	**7.5:1(5.0:1)**^c^
2	Ac	FeCl_3_	Et_2_O	85	7.5:1
3	Ac	FeCl_2_	Et_2_O	16	4.3:1
4	Ac	Fe(acac)_3_	toluene	94	3.9:1
5	Ac	Fe(acac)_3_	THF	90	2.2:1
6	Me	Fe(acac)_3_	Et_2_O	36	17:1
7	Piv	Fe(acac)_3_	Et_2_O	85	4.7:1
8^d^	Ac	Fe(acac)_3_	Et_2_O	92	7.5:1
9^e^	Ac	Fe(acac)_3_	Et_2_O	96	3.5:1
10^f^	Ac	–	Et_2_O	<5	–
11^g^	Ac	–	Et_2_O	<5	–

a Reaction conditions: **1a** (0.2 mmol), **2a** (0.25 mmol), Fe catalyst (5.0 mol %), solvent (1.0 mL), −20 °C, 20 min. Yields and *E*/*Z* ratios were determined by ^1^H NMR analysis of crude mixture with CH_2_Br_2_ as internal standards.

b Isolated yield in parentheses.

c *E*/*Z* ratio after isolation via chromatography in parentheses.

d 0 °C.

e 10 mol % of tetramethylethylenediamine (TMEDA) was added.

f No iron catalyst was added.

g Run with Pd(OAc)_2_ or CuI that would correspond to 0.1 wt % of the Fe(acac)_3_ (5 mol %) used.

We first explored the scope of α-allenyl acetates (Scheme 2). The α-phenyl-allenyl acetate (**1b**) gave the desired product **3ba** in 84% yield and excellent stereoselectivity (*E*/*Z* = 14:1). With the electron withdrawing *p*-chlorophenyl in the α-position (**1c**), the corresponding 1,3-diene was obtained in 92% yield (*E*/*Z* = 4.9:1). α-Allenyl acetate **1d** bearing a *m*-methoxyphenyl group in the α-position gave **3da** in 92% yield with a moderate *E*/*Z* ratio (4.1:1). α-Naphthyl-allenyl acetate (**1e**) was also a suitable substrate, which afforded 1,3-diene **3ea** in an almost quantitative yield with an *E*/*Z* ratio of 7.3:1. The unsubstituted α-allenyl acetate **1f** delivered 2-benzyl-1,3-butadiene (**3fa**) in 72% yield in 1.0 mmol scale. α-(*n*-Pentyl) substituted α-allenyl acetate was also a feasible substrate (**1g**), providing **3ga** in 84% yield and *E*/*Z* = 4.9:1. Trisubstituted allenic acetates with R^1^ = Me, R^2^ = Me (**1h** and **1i**) reacted successfully with **2a** to afford the corresponding dienes **3ha** and **3ia** in 84% and 89% yield, respectively. Sterically hindered tetrasubstituted α-allenyl acetates (**1j** to **1n**, R^1^, R^2^ = −(CH_2_)_*n*_–, *n* = 4 or 5) were also suitable substrates for this reaction and furnished the 1,3-dienes (**3ja** to **3na**) in yields from 59% to 91%. Notably, the reaction conditions were compatible with a Boc-protected (Boc = *tert*-butyloxylcarbonyl) cyclic amine, and 1,3-diene **3oa** was obtained in 40% yield.

**Scheme 2:**
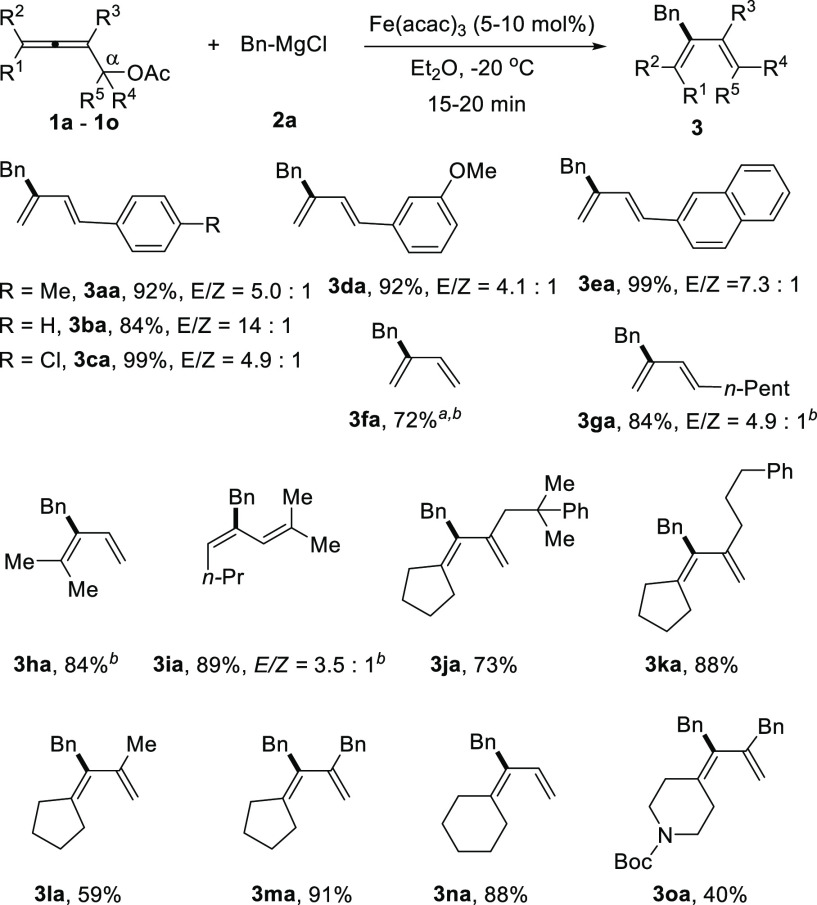
Scope of α-Allenyl Esters Butanoate instead of acetate substrate was used. With 1.0 mmol of **1**.

Next, the scope of Grignard reagents was investigated (Scheme 3). Methyl magnesium bromide (**2b**) was a reactive nucleophile for this reaction and afforded **3ab** in 90% yield with an *E*/*Z* ratio of 8.2:1. *n*-Butyl magnesium chloride (**2c**) bearing β-hydrogen atoms afforded the desired product in 63% yield and good *E*/*Z* ratio (10:1). Sterically hindered (2-methyl-2-phenylpropyl)magnesium chloride (**2d**) was also an applicable substrate, and the coupling product **3fd** from **1f** was isolated in 78% yield. (3-Phenylpropyl)magnesium chloride (**2e**) was successfully coupled with **1i**, **1k**, and **1l**, giving the corresponding products **3ie**, **3ke**, and **3le** in 64–92% yields in the presence of 1.0 mol % of Fe(acac)_3_. However, representative phenyl magnesium bromide (**2f**), ethyl magnesium bromide (**2g**), and α,α′-dioxo-ethyl magnesium bromide (**2h**) failed to give the desired products in practically useful yields with **1a**.^16^

**Scheme 3:**
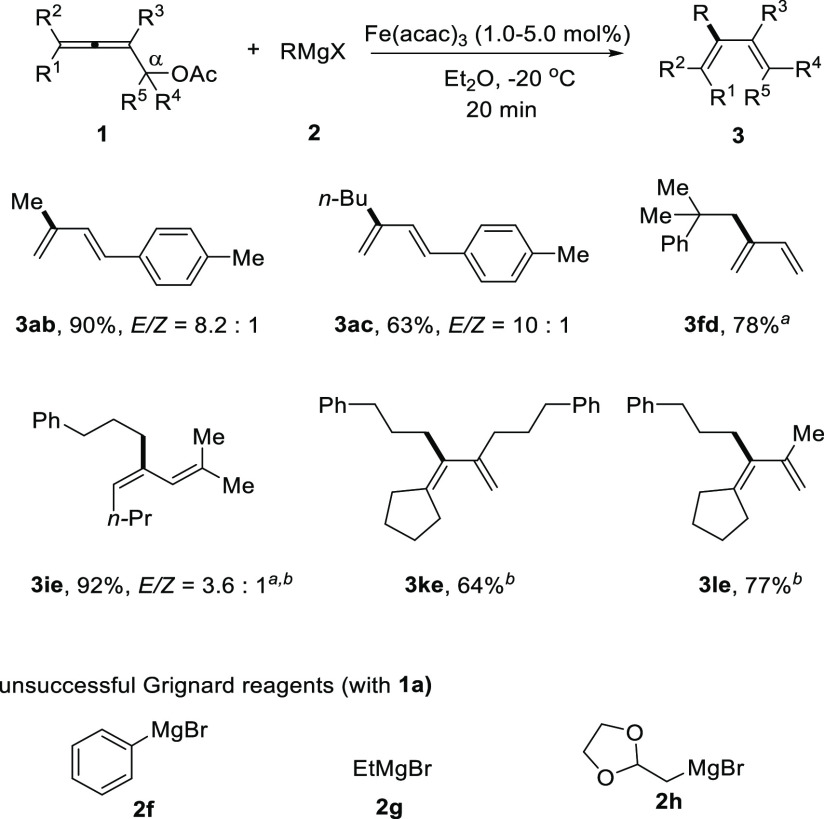
Scope of Grignard Reagent With 1.0 mmol of **1**. With 1.0 mol % of Fe(acac)_3_.

Based on these results and our previous work on iron catalysis, a plausible mechanism of this iron-catalyzed 1,3-diene synthesis reaction is proposed (Scheme 4). The addition of Grignard reagent **2** to the solution of the precatalyst Fe(acac)_3_ forms a reduced organoiron intermediate (Bn-[Fe]^*n*^MgX), whose exact structure is still unclear. This catalytically active species attacks α-allenyl acetate **1** through a *syn* or *anti* S_N_2′ pathway to form intermediate **int A** (oxidative addition). The latter intermediate would predominantly be of *E* configuration, since the R group in the α-position would prefer to be anti to the allene moiety. Subsequent reductive elimination would deliver **3** and regenerate the catalyst. The preferred *anti* conformation of the allene part and the *a*-substituent R results in the *E* configuration of C_α_=C_1_ independent of whether *syn* or *anti* S_N_2′ displacement occurs (Scheme 4). In the reaction with an α-allenyl acetate that has a substituent (R) in the 3-position, the S_N_2′ attack by Bn-[Fe]^*n*^MgX will occur from the face that avoids steric compulsion between the C_3_–R group and Bn-[Fe]^*n*^MX. The energetically favored pathway would lead to *E* configuration of C_2_=C_3_ (Scheme 5).

**Scheme 4:**
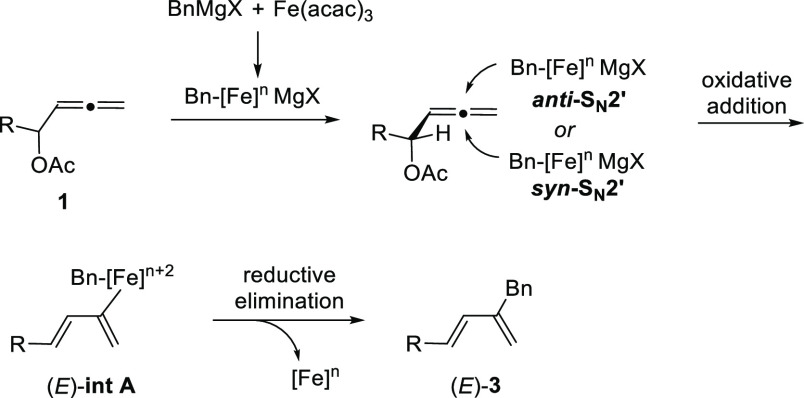
Proposed Reaction Mechanism

**Scheme 5:**
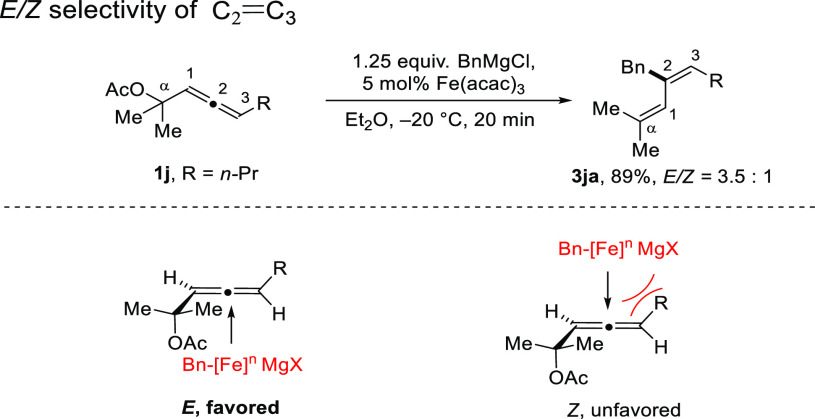
Conformational Analysis for *E*/*Z* Stereoselectivity of C_2_=C_3_

In summary, a simple and efficient approach to 1,3-dienes was realized through iron-catalyzed C–C bond coupling between α-allenyl acetates and Grignard reagents. A wide range of mono-, di-, tri-, and tetrasubstituted α-allenic acetates were applied, which led to the formation of structurally diverse 1,3-dienes. The reaction was associated with mild reaction conditions, high reactivity, good functional group compatibility, and easy scale up.

## Data Availability

The data underlying this study are available in the published article and its online Supporting Information.

## Supporting Information Available

Experimental procedures and spectral data for all new compounds (PDF)
