# Supplementary material for: Iron-Catalyzed Cross-Coupling of α-Allenyl Esters with Grignard Reagents for the Synthesis of 1,3-Dienes
Source: Org Lett. 2023 Jan 4;25(1):120–4. doi: 10.1021/acs.orglett.2c03916 (PMC9841610; doi:10.1021/acs.orglett.2c03916)
Supplement: Supplementary file 1 — ol2c03916_si_001.pdf [file ol2c03916_si_001.pdf]

Supporting Information

Iron-Catalyzed Cross-Coupling of α-Allenyl Esters with Grignard Reagents for the Synthesis of 1,3-Dienes

Wei-Jun Kong,[‡,*] Simon N. Kessler,[‡] Haibo Wu and Jan-E. Bäckvall*

Department of Organic Chemistry, Arrhenius Laboratory, Stockholm University, 10691 Stockholm, Sweden

Contents

1. General Information S2

2. General Procedure for Iron-Catalyzed Cross-Coupling of α-Allenyl Esters with Grignard Reagents S2

3. Control Experiments..S15

4. References S15

5. NMR Spectra..S16

1. General Information

Unless otherwise noted, all reagents were used as received from commercial suppliers. Dry solvents were obtained from commercial sources, from a VAC™ drying system or dried over molecular sieves. $Fe(acac)_3$ was purchased from Sigma-Aldrich (Prod. Nr.: 517003, Lot Nr.: MKBS7930V with a Cu content of 0.5 ppm). The starting material α-allenol esters were synthesized according to previous literature.[1] All reactions were conducted in dry flasks under argon atmosphere. Reactions were monitored using Merck silica gel 60 F254 plates (TLC analysis). Flash column chromatography was carried out with 60Å (particle size 35 - 70 μm) silica gel. ^{1}H-/^{13}C-NMR experiments were performed on a Bruker NMR (400/100 MHz) or (500/125 MHz) at room temperature. ^{19}F-NMR experiments were performed on a 400 MHz Bruker NMR (377 MHz). Chemical shifts (δ) are reported in parts per million (ppm) relative to the $CDCl_3$ peaks (δ(H) = 7.26 and δ(C) = 77.0 ppm). Coupling constants (*J*) are reported in Hertz (Hz). The following abbreviations were used to explain multiplicities: s = singlet, d = doublet, t = triplet, q = quartet, m = multiplet, and br = broad. HRMS were recorded on a Bruker MicroTOF spectrometer equipped with an ESI or APCI as ion sources.

2. General Procedure for Iron-Catalyzed Cross-Coupling of α-Allenyl Esters with Grignard Reagents

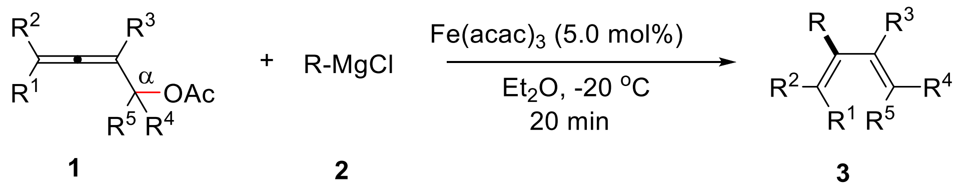

α-Allenol esters **1** (0.2 mmol) and $Fe(acac)_3$ (3.5 mg, 5.0 mol%) were added to a 10 mL dried vial. The system was vacuumed and refilled with argon thrice, followed by the addition of 1.0 mL of diethyl ether at -20 °C. Grignard reagent **2** (0.25 mmol) in ether was added dropwise to the mixture. After being stirred for 20 min at - 20 °C, the

reaction was quenched with 5% citric acid (0.5 ml). The residue was treated with H_2O (10 mL) and extracted with ethyl acetate (3×10 mL). The organic phase was dried over Na_2SO_4 and concentrated under vacuum. The crude mixtures were purified by flash column chromatography over silica gel to give the desired products.

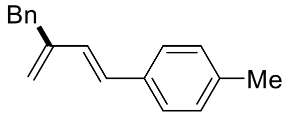

3aa

The general procedure was followed using **1a** (40.5 mg, 0.2 mmol) and **2a** (125.0 μL, 0.25 mmol, 2.0 M) in the presence of catalytic amount of $Fe(acac)_3$ (3.5 mg, 5.0 mol%) in 1.0 mL of added Et_2O at -20 °C for 20 min. Purification by column chromatography on silica gel (EtOAc /pentane = 1 : 100) yielded **3aa** (43.1 mg, 92%, *E/Z* = 5.0 : 1) as colorless liquid. **^{1}H NMR** (400 MHz, $CDCl_3$) δ 7.52 – 7.05 (m, 9H), 6.86 (d, *J* = 16.3 Hz, 1H), 6.60 (d, *J* = 16.3 Hz, 1H), 5.30 (d, *J* = 1.9 Hz, 1H), 4.97 (d, *J* = 1.9 Hz, 1H), 3.70 (s, 2H), 2.35 (s, 3H); **^{13}C NMR** (100 MHz, $CDCl_3$) δ 145.1, 139.5, 137.4, 134.4, 129.6, 129.3, 129.0, 128.9, 128.3, 126.4, 126.0, 118.1, 38.6, 21.2; **HRMS (ESI)**: m/z calcd. for $C_{18}H_{18}Na$ $[M+Na]^+$: 257.1301, found: 257.1302. The analytical data correspond with those reported in the literature.[2]

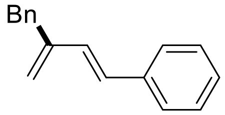

3ba

The general procedure was followed using **1b** (94.1 mg, 0.5 mmol) and **2a** (625.0 μL, 0.625 mmol , 1.0 M) in the presence of catalytic amount of $Fe(acac)_3$ (8.8 mg, 5.0 mol%) in 5.0 mL of added Et_2O at -20 °C for 15 min. Purification by column chromatography on silica gel (diethyl ether/pentane = 1 : 100) yielded **3ba** (91.6 mg, 83%, *E/Z* = 14.0 : 1) as colorless liquid. **^{1}H NMR** (400 MHz, $CDCl_3$) δ 7.51 – 7.19 (m, 10H), 6.93 (dd, *J* = 16.3, 6.0 Hz, 1H), 6.66 (dd, *J* = 16.3, 6.0 Hz, 1H), 5.36 (d, *J* = 4.4 Hz, 1H), 5.04 (d, *J* = 4.4 Hz, 1H), 3.74 (d, *J* = 4.3 Hz, 2H). (distinct minor isomer peaks) δ 6.46 (dd, *J* =

12.3, 5.0 Hz, 1H), 6.13 (dd, *J* = 12.3, 5.0 Hz, 1H). **^{13}C NMR** (100 MHz, $CDCl_3$) δ 145.0, 139.4, 137.2, 130.6, 129.1, 128.8, 128.5, 128.3, 127.5, 126.4, 126.1, 118.7, 38.7. The analytical data corresponds with those reported in the literature.[2]

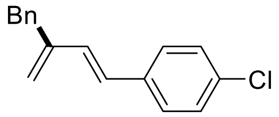

3ca

The general procedure was followed using **1c** (44.4 mg, 0.2 mmol) and **2a** (125.0 µL, 0.25 mmol, 2.0 M) in the presence of catalytic amount of $Fe(acac)_3$ (3.5 mg, 5.0 mol%) in 1.0 mL of added Et_2O at -20 °C for 20 min. Purification by column chromatography on silica gel (EtOAc/pentane = 1 : 50) yielded **3ca** (50.2 mg, 99%, , *E/Z* = 4.9 : 1) as colorless liquid. **^{1}H NMR** (400 MHz, $CDCl_3$) δ 7.41 – 7.21 (m, 9H), 6.89 (dd, *J* = 16.3, 0.7 Hz, 1H), 6.59 (d, *J* = 16.3 Hz, 1H), 5.37 (d, *J* = 1.7 Hz, 1H), 5.02 (d, *J* = 1.9 Hz, 1H), 3.72 (s, 2H). **^{13}C NMR** (100 MHz, $CDCl_3$) δ 144.7, 139.2, 135.7, 133.0, 131.2, 128.8, 128.7, 128.4, 127.7, 127.6, 126.1, 119.2, 38.6. **HRMS (ESI)**: m/z calcd. for $C_{17}H_{15}ClNa$ $[M+Na]^+$: 277.0754, found: 277.0752.

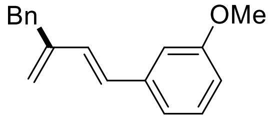

3da

The general procedure was followed using **1d** (43.6 mg, 0.2 mmol) and **2a** (125.0 µL, 0.25 mmol, 2.0 M) in the presence of catalytic amount of $Fe(acac)_3$ (3.5 mg, 5.0 mol%) at -20 °C in 1.0 mL of added Et_2O for 20 min. Purification by column chromatography on silica gel (EtOAc/pentane = 1 : 20) yielded **3da** (46.1 mg, 92%, *E/Z* = 4.1 : 1) as colorless liquid. **^{1}H NMR** (400 MHz, $CDCl_3$) δ 7.38 – 7.14 (m, 6H), 7.06 – 6.88 (m, 3H), 6.86 – 6.79 (m, 1H), 6.63 (d, *J* = 16.3 Hz, 1H), 5.36 (d, *J* = 1.9 Hz, 1H), 5.04 (d, *J* = 1.7 Hz, 1H), 3.85 (s, 3H), 3.73 (s, 2H). **^{13}C NMR** (100 MHz, $CDCl_3$) δ 159.9, 145.0,

139.4, 138.8, 131.0, 129.6, 129.0, 128.9, 128.4, 126.2, 119.3, 118.9, 113.3, 111.7, 55.3, 38.7. **HRMS (ESI)**: m/z calcd. for $C_{18}H_{18}ONa$ [M+Na]$^+$: 273.1250, found: 273.1258.

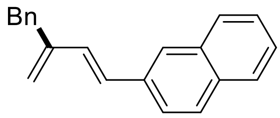

3ea

The general procedure was followed using **1e** (47.7mg, 0.2 mmol) and **2a** (125.0 μL, 0. 25 mmol, 2.0 M) in the presence of catalytic amount of Fe(acac)$_3$ (3.5 mg, 5.0 mol%) in 1.0 mL of added Et_2O at -20 °C for 20 min. Purification by column chromatography on silica gel (EtOAc/pentane = 1 : 50) yielded **3ea** (54.0 mg, 99%,, *E/Z* = 7.3 : 1) as colorless liquid. **^{1}H NMR** (400 MHz, $CDCl_3$) δ 7.85 – 7.74 (m, 3H), 7.66 (dd, *J* = 8.7, 1.8 Hz, 2H), 7.57 – 7.42 (m, 2H), 7.40 – 7.31 (m, 4H), 7.30 – 7.20 (m, 1H), 7.05 (d, *J* = 16.2 Hz, 1H), 6.82 (d, *J* = 16.2 Hz, 1H), 5.43 – 5.38 (m, 1H), 5.07 (d, *J* = 1.5 Hz, 1H), 3.78 (d, *J* = 1.2 Hz, 2H). **^{13}C NMR** (100 MHz, $CDCl_3$) δ 145.0, 139.4, 134.7, 133.6, 133.0, 130.9, 129.1, 128.9, 128.4, 128.2, 127.9, 127.6, 126.6, 126.2, 126.1, 125.8, 123.5, 118.9, 38.7. **HRMS (ESI)**: m/z calcd. for $C_{21}H_{19}$ [M+H]$^+$: 271.1481, found: 271.1472.

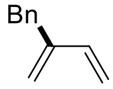

3fa

The general procedure was followed using **1f** (140.2 mg, 1.0 mmol) and **2a** (1.25 mL, 1.25 mmol, 1.0 M) in the presence of catalytic amount of Fe(acac)$_3$ (17.7 mg, 5.0 mol%) in 5.0 mL of added Et_2O at -20 °C for 15 min. Purification by column chromatography on silica gel (diethyl ether/pentane = 1 : 100) yielded **3fa** (103.4 mg, 72%) as colorless liquid. **^{1}H NMR** (400 MHz, $CDCl_3$) δ 7.35 – 7.28 (m, 2H), 7.25 – 7.17 (m, 3H), 6.46 (dd, *J* = 17.6, 10.8 Hz, 1H), 5.31 – 5.22 (m, 1H), 5.19 (dt, *J* = 1.2, 0.6 Hz, 1H), 5.09 (d, *J* = 10.8 Hz, 1H), 4.96 – 4.91 (m, 1H), 3.58 (s, 2H). **^{13}C NMR** (100 MHz, $CDCl_3$) δ

145.2, 139.5, 138.5, 128.8, 128.3, 126.0, 118.2, 114.3, 38.1. The analytical data correspond with those reported in the literature.[3]

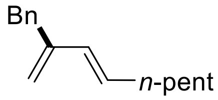

3ga

The general procedure was followed using **1g** (91.1 mg, 0.5 mmol) and **2a** (625.0 μL, 0.625 mmol, 1.0 M) in the presence of catalytic amount of $Fe(acac)_3$ (8.8 mg, 5.0 mol%) in 2.5 mL of added Et_2O at -20 °C for 15 min. Purification by column chromatography on silica gel (diethyl ether/pentane = 1 : 100) yielded **3ga** (101.0 mg, 94%, *E/Z* = 4.9 : 1) as colorless liquid. **^{1}H NMR** (400 MHz, $CDCl_3$) δ 7.39 – 7.13 (m, 5H), 6.16 (d, *J* = 15.6 Hz, 1H), 5.77 (dt, *J* = 15.8, 6.9 Hz, 1H), 5.09 (d, *J* = 2.0 Hz, 1H), 4.81 (d, *J* = 2.0 Hz, 1H), 3.58 (s, 2H), 2.14 – 2.04 (m, 2H), 1.44 – 1.20 (m, 6H), 0.91 (t, *J* = 7.0 Hz, 3H). **^{13}C NMR** (100 MHz, $CDCl_3$) δ 145.2, 139.8, 131.6, 131.5, 128.8, 128.2, 125.9, 115.5, 38.9, 32.8, 31.3, 29.0, 22.5, 14.0. **HRMS (ESI)**: m/z calcd. for $C_{16}H_{23}[M+H]^+$: 215.1794, found: 215.1785.

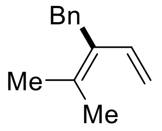

3ha

The general procedure was followed using **1h** (140.2 mg, 1.0 mmol) and **2a** (1.25 mL, 1.25 mmol, 1.0 M) in the presence of catalytic amount of $Fe(acac)_3$ (17.7 mg, 5.0 mol%) in 5.0 mL of added Et_2O at -20 °C for 15 min. Purification by column chromatography on silica gel (diethyl ether/pentane = 1 : 50) yielded **3ha** (143.9 mg, 84%) as colorless liquid. **^{1}H NMR** (400 MHz, $CDCl_3$) δ 7.35 – 7.26 (m, 2H), 7.23 – 7.16 (m, 3H), 6.90 (dd, *J* = 17.3, 11.1 Hz, 1H), 5.11 (d, *J* = 17.3 Hz, 1H), 5.00 (d, *J* = 11.1 Hz, 1H), 3.71 (s, 2H), 1.98 (s, 3H), 1.87 (s, 3H). **^{13}C NMR** (100 MHz, $CDCl_3$) δ 140.7, 134.8, 133.9, 129.7, 128.2, 127.8, 125.5, 112.2, 33.6, 22.0, 20.4. **HRMS (ESI)**: m/z calcd. for $C_{13}H_{17}$ $[M+H]^+$: 173.1325, found: 173.1319.

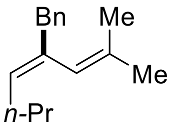

***E*-3ia**

The general procedure was followed using **1i** (182.3 mg, 1.0 mmol) and **2a** (1.25 mL, 1.25 mmol ,1.0 M) in the presence of catalytic amount of Fe(acac)$_3$ (17.7 mg, 5.0 mol%) in 5.0 mL of added Et$_2$O at -20 °C for 15 min. Purification by column chromatography on silica gel (pentane) yielded ***E*-3ia** (147.0 mg, 69%) as colorless liquid. **^{1}H NMR** (400 MHz, CDCl$_3$) δ 7.33 – 7.24 (m, 2H), 7.24 – 7.16 (m, 3H), 5.49 (s, 1H), 5.31 (td, *J* = 7.1, 1.0 Hz, 1H), 3.35 (s, 2H), 1.97 – 1.87 (m, 2H), 1.73 (d, *J* = 1.4 Hz, 3H), 1.48 (d, *J* = 1.1 Hz, 3H), 1.41 (dt, *J* = 14.8, 7.4 Hz, 2H), 0.92 (t, *J* = 7.4 Hz, 3H). **^{13}C NMR** (100 MHz, CDCl$_3$) δ 140.6, 136.6, 134.4, 128.9, 128.5, 128.0, 125.7, 123.3, 44.8, 31.4, 25.3, 22.7, 19.5, 14.0. **HRMS (ESI)**: m/z calcd. for $C_{16}H_{23}$ [M+H]$^+$: 215.1794, found: 215.1803.

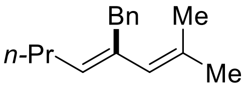

***Z*-3ia**

The general procedure was followed using **1i** (182.3 mg, 1.0 mmol) and **2a** (1.25 mL, 1.25 mmol, 1.0 M) in the presence of catalytic amount of Fe(acac)$_3$ (17.7 mg, 5.0 mol%) in 5.0 mL of added Et$_2$O at -20 °C for 15 min. Purification by column chromatography on silica gel (pentane) yielded ***Z*-3ia** (43.0 mg, 20%) as colorless liquid. **^{1}H NMR** (400 MHz, CDCl$_3$) δ 7.32 – 7.24 (m, 2H), 7.23 – 7.15 (m, 3H), 5.59 – 5.54 (m, 1H), 5.44 (t, *J* = 7.3 Hz, 1H), 3.47 (s, 2H), 2.20 (q, *J* = 7.3 Hz, 2H), 1.73 (dd, *J* = 7.1, 1.2 Hz, 6H), 1.48 (q, *J* = 7.4 Hz, 2H), 0.97 (t, *J* = 7.4 Hz, 3H). **^{13}C NMR** (100 MHz, CDCl$_3$) δ 140.4, 135.3, 133.1, 130.4, 128.4, 128.2, 127.5, 125.6, 37.0, 30.5, 26.4, 23.1, 19.4, 14.0. **HRMS (ESI)**: m/z calcd. for $C_{16}H_{23}$ [M+H]$^+$: 215.1794, found: 215.1758.

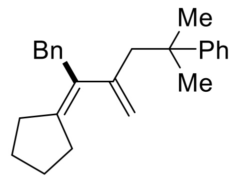

3ja

The general procedure was followed using **1i** (142.2 mg, 0.5 mmol) and **2a** (0.75 mL, 0.75 mmol, 1.0 M) in the presence of catalytic amount of $Fe(acac)_3$ (17.7 mg, 10.0 mol%) in 5.0 mL of added Et_2O at -20 °C for 15 min. Purification by column chromatography on silica gel (diethyl ether/pentane = 1 : 100 to 1 : 50) yielded **3ja** (115.0 mg, 73%) as colorless liquid. **^{1}H NMR** (400 MHz, $CDCl_3$) δ 7.43 – 7.13 (m, 10H), 4.85 (s, 1H), 4.76 (s, 1H), 3.26 (s, 2H), 2.54 (s, 2H), 2.28 (t, *J* = 6.7 Hz, 2H), 2.14 (t, *J* = 7.0 Hz, 2H), 1.67 – 1.53 (m, 4H), 1.43 – 1.38 (m, 6H). **^{13}C NMR** (100 MHz, $CDCl_3$) δ 149.2, 147.0, 141.1, 140.8, 131.9, 128.6, 128.0, 127.7, 126.0, 125.5, 125.4, 118.4, 49.2, 38.3, 37.7, 33.0, 30.8, 29.3, 26.8, 26.2. **HRMS (ESI)**: m/z calcd. for $C_{25}H_{30}Na$ $[M+Na]^+$: 353.2240, found: 353.2236.

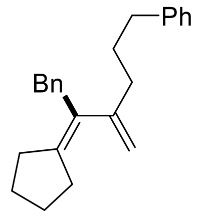

3ka

The general procedure was followed using **1k** (142.2 mg, 0.5 mmol) and **2a** (0.75 mL, 0.75 mmol, 1.0 M) in the presence of catalytic amount of $Fe(acac)_3$ (12.4 mg, 7.0 mol%) in 5.0 mL of added Et_2O at -20 °C for 15min. Purification by column chromatography on silica gel (diethyl ether/pentane = 1 : 50) yielded **3ka** (139.3 mg, 88%) as colorless liquid. **^{1}H NMR** (400 MHz, $CDCl_3$) δ 7.37 – 7.13 (m, 10H), 4.97 – 4.92 (m, 1H), 4.72 (d, *J* = 2.4 Hz, 1H), 3.47 (s, 2H), 2.63 – 2.55 (m, 2H), 2.40 (q, *J* = 6.0 Hz, 4H), 2.13 (t, *J* = 7.6 Hz, 2H), 1.79 – 1.60 (m, 6H). **^{13}C NMR (**100 MHz, $CDCl_3$) δ 145.0, 142.7, 141.3, 140.6, 131.2, 128.6, 128.5, 128.3, 128.1, 125.7, 125.6, 113.4, 38.4, 35.8, 35.1,

32.6, 30.8, 29.7, 27.0, 26.4. **HRMS (ESI)**: m/z calcd. for $C_{24}H_{29}$ $[M+H]^+$: 317.2264, found: 317.2257.

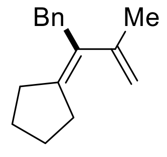

3la

The general procedure was followed using **1k** (90.1 mg, 0.5 mmol) and **2a** (0.75 mL, 0.75 mmol, 1.0 M) in the presence of catalytic amount of $Fe(acac)_3$ (13.2 mg, 7.5 mol%) in 5.0 mL of added Et_2O at -20 °C for 15min. Purification by column chromatography on silica gel (pentane) yielded **3la** (63.0 mg, 59%) as colorless liquid. **^{1}H NMR** (400 MHz, $CDCl_3$) δ 7.60 – 7.01 (m, 5H), 4.94 – 4.89 (m, 1H), 4.66 (s, 1H), 3.51 (s, 2H), 2.38 (t, J = 6.5 Hz, 4H), 1.77 – 1.63 (m, 7H). **^{13}C NMR** (100 MHz, $CDCl_3$) δ 146.2, 140.6, 140.1, 131.8, 128.5, 128.1, 125.6, 113.7, 38.7, 32.3, 30.8, 27.0, 26.4, 22.4. **HRMS (ESI)**: m/z calcd. for $C_{16}H_{21}$ $[M+H]^+$: 213.1638, found: 213.1647.

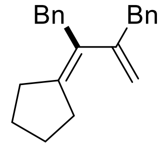

3ma

The general procedure was followed using **1k** (128.2 mg, 0.5 mmol) and **2a** (0.75 mL, 0.75 mmol, 1.0 M) in the presence of catalytic amount of $Fe(acac)_3$ (12.4 mg, 7.0 mol%) in 5.0 mL of added Et_2O at -20 °C for 15min. Purification by column chromatography on silica gel (diethyl ether/pentane = 1 : 50) yielded **3ma** (130.9 mg, 91%) as colorless liquid. **^{1}H NMR** (400 MHz, $CDCl_3$) δ 7.36 – 7.11 (m, 10H), 4.82 – 4.79 (m, 1H), 4.77 – 4.73 (m, 1H), 3.49 (s, 2H), 3.35 (s, 2H), 2.36 (t, J = 7.1 Hz, 2H), 2.27 (t, J = 7.1 Hz, 2H), 1.73 – 1.66 (m, 2H), 1.61 – 1.52 (m, 2H). **^{13}C NMR** (100 MHz, $CDCl_3$) δ 149.7, 141.5, 140.5, 139.7, 131.0, 129.3, 128.7, 128.13, 128.06, 125.9, 125.7, 114.9, 42.1, 38.7,

32.4, 30.7, 26.9, 26.4. **HRMS (ESI)**: m/z calcd. for $C_{22}H_{25}[M+H]^+$: 289.1951, found: 289.1941.

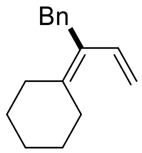

3na

The general procedure was followed using **1n** (36.0 mg, 0.2 mmol) and **2a** (125.0 µL, 0.25 mmol, 2.0 M) in the presence of catalytic amount of $Fe(acac)_3$ (3.5 mg, 5.0 mol%) in 1.0 mL of added Et_2O at -20 °C for 20 min. Purification by column chromatography on silica gel (EtOAc/pentane = 1: 200) yielded **3na** (37.3 mg, 88%) as colorless liquid. **^{1}H NMR** (400 MHz, $CDCl_3$) δ 7.35 – 7.29 (m, 2H), 7.27 – 7.19 (m, 3H), 5.56 (s, 1H), 5.01 (dt, *J* = 2.4, 1.2 Hz, 1H), 4.89 (s, 1H), 3.43 (s, 2H), 2.35 – 2.27 (m, 2H), 2.11 (t, *J* = 5.5 Hz, 2H), 1.56 (dd, *J* = 6.2, 3.0 Hz, 4H), 1.48 – 1.38 (m, 2H). **^{13}C NMR** (100 MHz, $CDCl_3$) δ 144.9, 143.1, 139.8, 128.9, 128.1, 125.9, 122.5, 114.0, 44.1, 37.6, 29.8, 28.6, 27.9, 26.7. **HRMS (ESI)**: m/z calcd. for $C_{16}H_{20}Na$ $[M+Na]^+$: 235.1456, found: 235.1457.

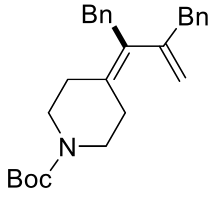

3oa

The general procedure was followed using **1o** (74.3 mg, 0.2 mmol) and **2a** (125.0 µL, 0.25 mmol, 2.0 M) in the presence of catalytic amount of $Fe(acac)_3$ (3.5 mg, 5.0 mol%) in 1.0 mL of added Et_2O at -20 °C for 20 min. Purification by column chromatography on silica gel (EtOAc/pentane = 1: 50) yielded **3na** (32.3 mg, 40%) as colorless liquid. **^{1}H NMR** (400 MHz, $CDCl_3$) δ 7.44 – 7.01 (m, 10H), 4.85 (s, 1H), 4.53 (d, *J* = 1.8 Hz, 1H), 3.53 (s, 2H), 3.44 – 3.33 (m, 4H), 3.13 – 3.05 (m, 2H), 2.35 (s, 2H), 2.21 – 2.11

(m, 2H), 1.48 (s, 9H). **^{13}C NMR** (100 MHz, $CDCl_3$) δ 154.7, 148.3, 140.0, 138.9, 133.3, 132.3, 129.3, 128.6, 128.2, 128.1, 126.1, 125.9, 114.9, 79.3, 44.8, 44.0, 42.8, 36.5, 31.1, 29.9, 28.4. HRMS (ESI): m/z calcd. for $C_{27}H_{33}NNaO_2$ $[M+Na]^+$: 426.2404, found: 426.2401.

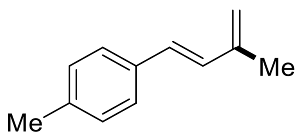

3ab

The general procedure was followed using **1a** (40.5 mg, 0.2 mmol) and MeMgBr **2b** (84.0 μL, 0.25 mmol, 3.0 M) in the presence of catalytic amount of $Fe(acac)_3$ (3.5 mg, 5.0 mol%) in 1.0 mL of added Et_2O at -20 °C for 20 min. Purification by column chromatography on silica gel (EtOAc/pentane = 1: 100) yielded **3ab** (28.5 mg, 90%, *E/Z* = 8.2 : 1) as colorless liquid. **^{1}H NMR** (400 MHz, $CDCl_3$) δ 7.42 – 7.32 (m, 2H), 7.20 – 7.15 (m, 2H), 6.89 (d, *J* = 16.1 Hz, 1H), 6.56 (d, *J* = 16.1 Hz, 1H), 5.14 (dt, *J* = 1.4, 0.7 Hz, 1H), 5.12 – 5.07 (m, 1H), 2.39 (s, 3H), 2.02 (dd, *J* = 1.3, 0.7 Hz, 3H). **^{13}C NMR** (100 MHz, $CDCl_3$) δ 142.1, 137.2, 134.6, 130.7, 129.3, 128.6, 126.4, 116.8, 21.2, 18.6; **HRMS (ESI)**: m/z calcd. for $C_{12}H_{14}Na$ $[M+Na]^+$: 181.0988, found: 181.0999.

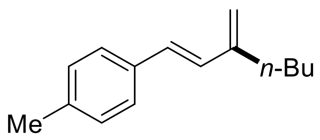

3ac

The general procedure was followed using **1a** (40.5 mg, 0.2 mmol) and **2b** (125.0 μL, 0.25 mmol, 2.0 M) in the presence of catalytic amount of $Fe(acac)_3$ (3.5 mg, 5.0 mol%) in 1.0 mL of added Et_2O at -20 °C for 20 min. Purification by column chromatography on silica gel (EtOAc/pentane = 1: 200) yielded **3ac** (25.2 mg, 63%, *E/Z* = 10.0 : 1) as colorless liquid. **^{1}H NMR** (400 MHz, $CDCl_3$) δ 7.36 (d, *J* = 8.0 Hz, 2H), 7.17 (d, *J* = 8.0 Hz, 2H), 6.80 (d, *J* = 16.3 Hz, 1H), 6.60 (d, *J* = 16.3 Hz, 1H), 5.15 (d, *J* = 2.0 Hz, 1H), 5.07 (d, *J* = 2.0 Hz, 1H), 2.40 - 2.33 (m, 5H), 1.58 (tt, *J* = 7.7, 5.5 Hz, 2H), 1.49 – 1.39 (m, 2H), 0.99 (t, *J* = 7.3 Hz, 3H). **^{13}C NMR** (100 MHz, $CDCl_3$) δ 146.5, 137.2,

134.7, 130.2, 129.3, 127.8, 126.3, 115.5, 31.8, 30.6, 22.7, 21.2, 14.0. **HRMS** (ESI): m/z calcd. for $C_{15}H_{21}$ [M+H]$^+$: 201.1638, found: 201.1650.

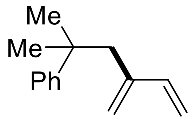

3fd

The general procedure was followed using **1f** (142.2 mg, 1.0 mmol) and **2e** (2.5 mL, 1.25 mmol, 0.5 M) in the presence of catalytic amount of Fe(acac)$_3$ (17.7 mg, 5.0 mol%) in 5.0 mL of added Et_2O at -20 °C for 15 min. Purification by column chromatography on silica gel (diethyl ether/pentane = 1: 200) yielded **3fd** (146.0 mg, 78%) as colorless liquid. ^{1}**H NMR** (400 MHz, $CDCl_3$) δ 7.44 – 7.37 (m, 2H), 7.36 – 7.29 (m, 2H), 7.23 – 7.16 (m, 1H), 6.28 (ddd, *J* = 17.5, 10.9, 0.8 Hz, 1H), 5.16 (ddt, *J* = 17.5, 1.0, 0.6 Hz, 1H), 5.07 (dq, *J* = 2.2, 0.7 Hz, 1H), 4.96 (dq, *J* = 10.9, 1.0 Hz, 1H), 4.63 – 4.61 (m, 1H), 2.55 (d, *J* = 0.9 Hz, 2H), 1.37 (s, 6H). ^{13}**C NMR** (100 MHz, $CDCl_3$) δ 149.6, 143.4, 140.3, 127.9, 126.0, 125.5, 118.8, 113.2, 44.9, 38.2, 28.8. **HRMS** (ESI): m/z calcd. for $C_{14}H_{18}Na$ [M+Na]$^+$: 209.1301, found: 209.1309.

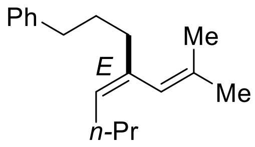

***E*-3ie**

The general procedure was followed using **1i** (182.3 mg, 1.0 mmol) and **2e** (1.98 mL, 1.25 mmol, 0.63 M) in the presence of catalytic amount of Fe(acac)$_3$ (3.5 mg, 1.0 mol%) in 5.0 mL of added Et_2O at -20 °C for 15 min. Purification by column chromatography on silica gel (pentane) yielded ***E*-3ie** (175.2 mg, 72%) as colorless liquid. ^{1}**H NMR** (500 MHz, $CDCl_3$) δ 7.33 – 7.26 (m, 2H), 7.21 – 7.17 (m, 3H), 5.56 – 5.52 (m, 1H), 5.23 (td, *J* = 7.1, 1.2 Hz, 1H), 2.63 – 2.56 (m, 2H), 2.09 (t, *J* = 7.5 Hz, 2H), 1.93 – 1.85 (m, 2H), 1.80 (d, *J* = 1.5 Hz, 3H), 1.74 – 1.65 (m, 2H), 1.58 (d, *J* = 1.3 Hz, 3H), 1.37 (h, *J* = 7.3 Hz, 2H), 0.91 (t, *J* = 7.4 Hz, 3H). ^{13}**C NMR** (125 MHz, $CDCl_3$) δ 142.9,

137.1, 134.1, 128.4, 128.2, 127.1, 125.5, 123.8, 38.0, 35.6, 31.3, 30.3, 25.4, 22.8, 19.7, 19.7, 14.0. **HRMS** (ESI): m/z calcd. for $C_{18}H_{27}$ $[M+H]^+$: 243.2107, found: 243.2117.

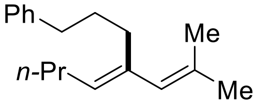

***Z*-3ie**

The general procedure was followed using **1i** (182.3 mg, 1.0 mmol) and **2e** (1.98 mL, 1.25 mmol, 0.63 M) in the presence of catalytic amount of $Fe(acac)_3$ (3.5 mg, 1.0 mol%) in 5.0 mL of added Et_2O at -20 °C for 15 min. Purification by column chromatography on silica gel (pentane) yielded **Z-3ie** (49.5 mg, 20%) as colorless liquid. **^{1}H NMR** (400 MHz, $CDCl_3$) δ 7.36 – 7.27 (m, 2H), 7.23– 7.17 (m, 3H), 5.64 – 5.59 (m, 1H), 5.24 (ddd, *J* = 8.2, 7.2, 1.3 Hz, 1H), 2.66 – 2.56 (m, 2H), 2.19 – 2.02 (m, 4H), 1.79 (dd, *J* = 4.1, 1.5 Hz, 6H), 1.75 – 1.64 (m, 2H), 1.48 – 1.37 (m, 2H), 0.95 (t, *J* = 7.3 Hz, 3H). **^{13}C NMR** (100 MHz, $CDCl_3$) δ 142.8, 137.0, 132.9, 129.0, 128.4, 128.2, 127.8, 125.6, 35.9, 30.8, 30.5, 30.1, 26.4, 23.2, 19.4, 14.0. **HRMS** (ESI): m/z calcd. for $C_{18}H_{27}$ $[M+H]^+$: 243.2107, found: 243.2084.

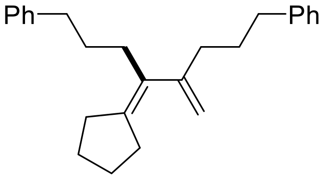

3ke

The general procedure was followed using **1k** (142.2 mg, 0.5 mmol) and **2e** (0.79 mL, 0.75 mmol, 0.95 M) in the presence of catalytic amount of $Fe(acac)_3$ (1.8 mg, 1.0 mol%) in 5.0 mL of added Et_2O at -20 °C for 15 min. Purification by column chromatography on silica gel (diethyl ether/pentane = 1: 40) yielded **3ke** (110.3 mg, 64%) as colorless liquid. **^{1}H NMR** (400 MHz, $CDCl_3$) δ 7.37 – 7.28 (m, 4H), 7.27 – 7.19 (m, 6H), 5.04 – 4.99 (m, 1H), 4.80 (d, *J* = 2.5 Hz, 1H), 2.64 (q, *J* = 8.0 Hz, 4H), 2.33 (t, *J* = 7.0 Hz, 2H), 2.26 (t, *J* = 7.3 Hz, 2H), 2.15 (q, *J* = 7.1 Hz, 4H), 1.76 – 1.56 (m, 8H). **^{13}C NMR** (100 MHz, $CDCl_3$) δ 150.2, 142.9, 142.7, 139.2, 132.4, 128.5, 128.4, 128.3, 128.2,

125.7, 125.6, 112.7, 35.8, 35.7, 35.0, 32.4, 31.5, 30.1, 29.9, 29.8, 26.9, 26.4. **HRMS** (ESI): m/z calcd. for $C_{26}H_{33}$ $[M+H]^+$: 345.2577, found: 345.2581.

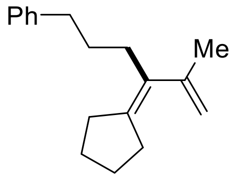

3le

The general procedure was followed using **1l** (90.1 mg, 0.5 mmol) and **2e** (0.78 mL, 0.625 mmol, 0.8 M) in the presence of catalytic amount of $Fe(acac)_3$ (1.8 mg, 1.0 mol%) in 5.0 mL of added Et_2O at -20 °C for 15 min. Purification by column chromatography on silica gel (pentane) yielded **3le** (92.4 mg, 77%) as colorless liquid. **^{1}H NMR** (400 MHz, $CDCl_3$) δ 7.38 – 7.17 (m, 5H), 4.97 (dt, J = 3.0, 1.5 Hz, 1H), 4.72 (dd, J = 2.7, 0.9 Hz, 1H), 2.66 – 2.59 (m, 2H), 2.29 (tt, J = 7.0, 1.4 Hz, 2H), 2.26 – 2.20 (m, 2H), 2.17 (t, J = 7.7 Hz, 2H), 1.79 (t, J = 1.2 Hz, 3H), 1.74 – 1.54 (m, 6H). **^{13}C NMR** (100 MHz, $CDCl_3$) δ 146.3, 142.8, 137.9, 132.9, 128.3, 128.2, 125.5, 113.1, 35.6, 32.1, 31.6, 30.1, 29.8, 26.9, 26.4, 22.2. **HRMS** (ESI): m/z calcd. for $C_{18}H_{25}$ $[M+H]^+$: 241.1951, found: 241.1948.

3. Control Experiments

Control experiments using low amounts of $Pd(OAc)_2$ or CuI in the absence of iron catalyst in attempted cross coupling.

A vial was charged with 0.35 mg metal salt $Pd(OAc)_2$ or CuI and 1.0 mL Et_2O, and then 0.1 mL of the resulting solution was added to another vial and 0.9 ml Et_2O was added to dilute the solution. By doing so, a solution of metal salt in Et_2O with a concentration of (0.035 mg/mL) was obtained. α-Allenol ester **1a** (0.2 mmol) and 0.1 mL of the solution of metal salt in Et_2O (0.035 mg/mL) was added to a 10 mL dry vial under argon and followed by the addition of 0.9 mL Et_20. Thus, the metal salt used in the reaction was 0.0035 mg, which corresponds to 0.1 wt.% of the catalyst loading of

$Fe(acac)_3$. Grignard reagent **2a** (0.125 μL, 0.25 mmol; 2M) was added dropwise to the mixture at -20 °C and the resulting mixture was stirred for 20 mins. The reaction was quenched with 5% citric acid (0.5 ml). The residue was treated with H_2O (10 mL) and extracted with ethyl acetate (3×10 mL). The organic phase was dried over Na_2SO_4 and concentrated under vacuum. 14 μL of CH_2Br_2 (0.20 mmol) was added to the residue as an internal standard. The crude ^1H NMR shows that the yield of coupling product **3aa** was <5% in both cases.

4. References

(1). a) Yang, B.; Zhu, C.; Qiu, Y.; Bäckvall, J.-E. Enzyme- and Ruthenium-Catalyzed Enantioselective Transformation of α-Allenic Alcohols into 2,3-Dihydrofurans. *Angew. Chem. Int. Ed.* **2016**, *55*, 5568-5572; b) Kessler, S. N.; Hundemer, F.; Bäckvall, J.-E. A Synthesis of Substituted α-Allenols via Iron-Catalyzed Cross-Coupling of Propargyl Carboxylates with Grignard Reagents. *ACS Catal.* **2016**, *6*, 7448-7451; c) Kessler, S. N.; Bäckvall, J.-E. Iron-catalyzed Cross-Coupling of Propargyl Carboxylates and Grignard Reagents: Synthesis of Substituted Allenes. *Angew. Chem. Int. Ed.* **2016**, *55*, 3734-3738.

(2). Zheng, C.; Wang, D.; Stahl, S. S. Catalyst-Controlled Regioselectivity in the Synthesis of Branched Conjugated Dienes via Aerobic Oxidative Heck Reactions. *J. Am. Chem. Soc.* **2012**, *134*, 16496-16499.

(3). Barluenga, J.; Rodríguez, F.; Álvarez-Rodrigo, L.; Fañanás, F. J. Zirconium-Mediated Cross-Coupling of Terminal Alkynes and Vinyl Bromides: Selective Synthesis of Cyclobutene and 1,3-Diene Derivatives. *Chem. Eur. J.* **2004**, *10*, 101-108.

5. NMR Spectra

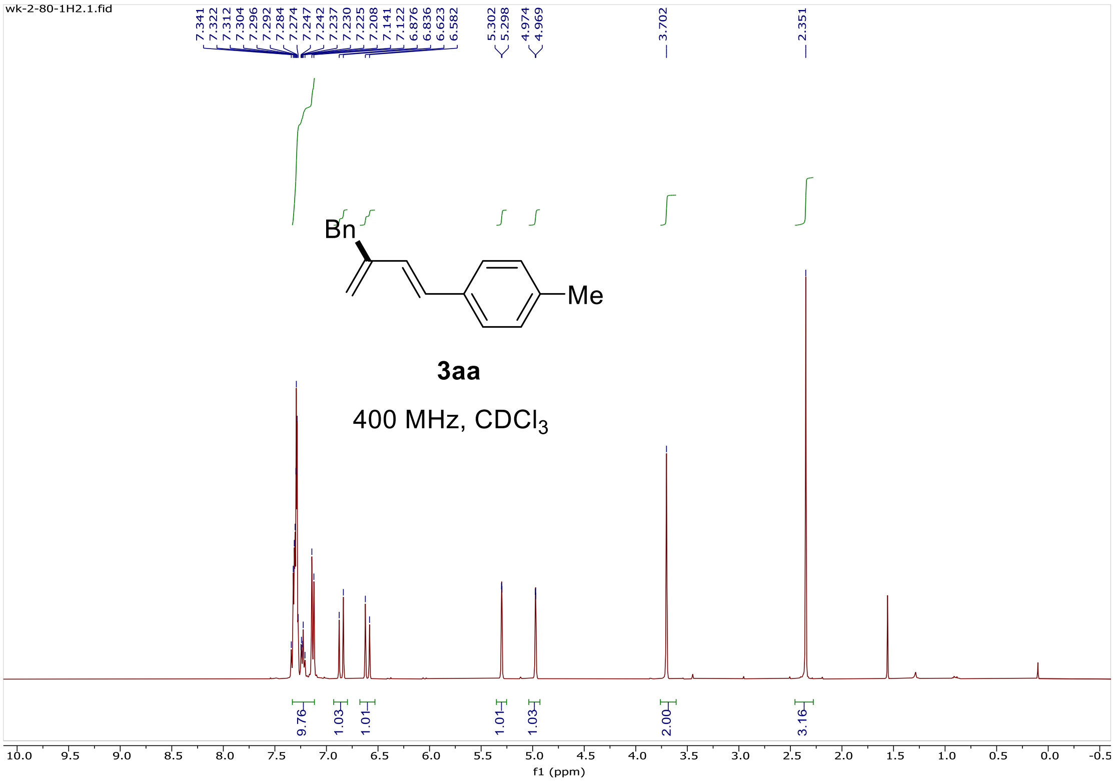

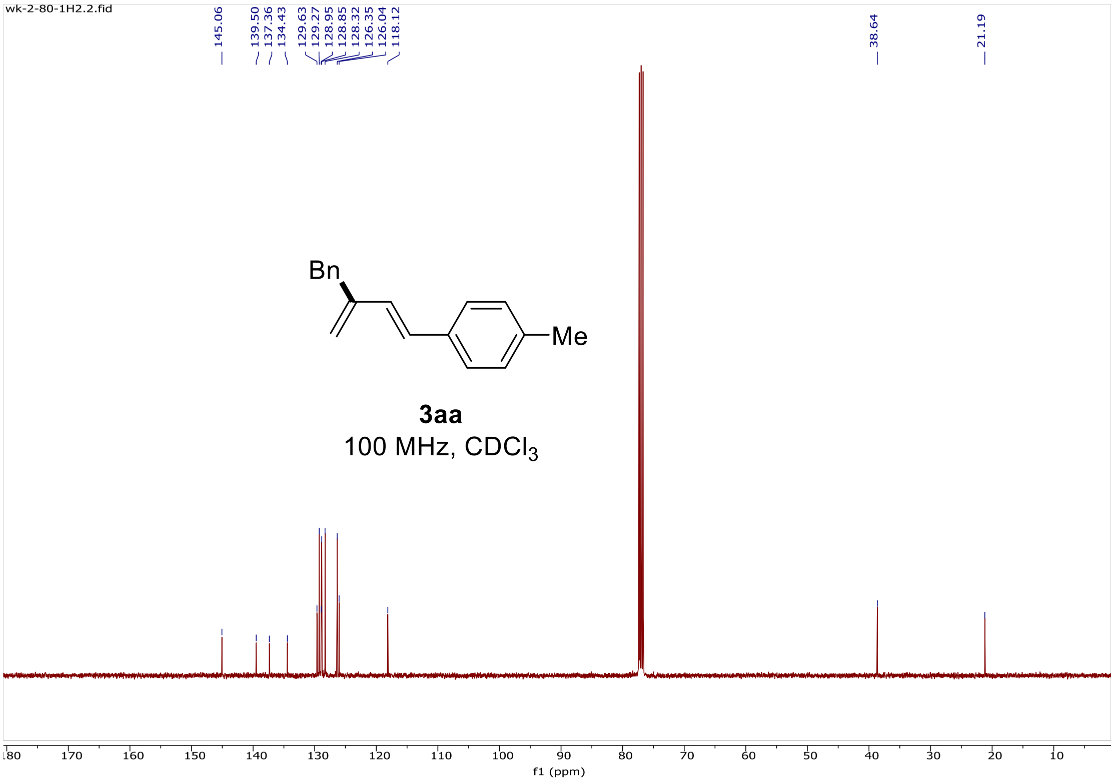

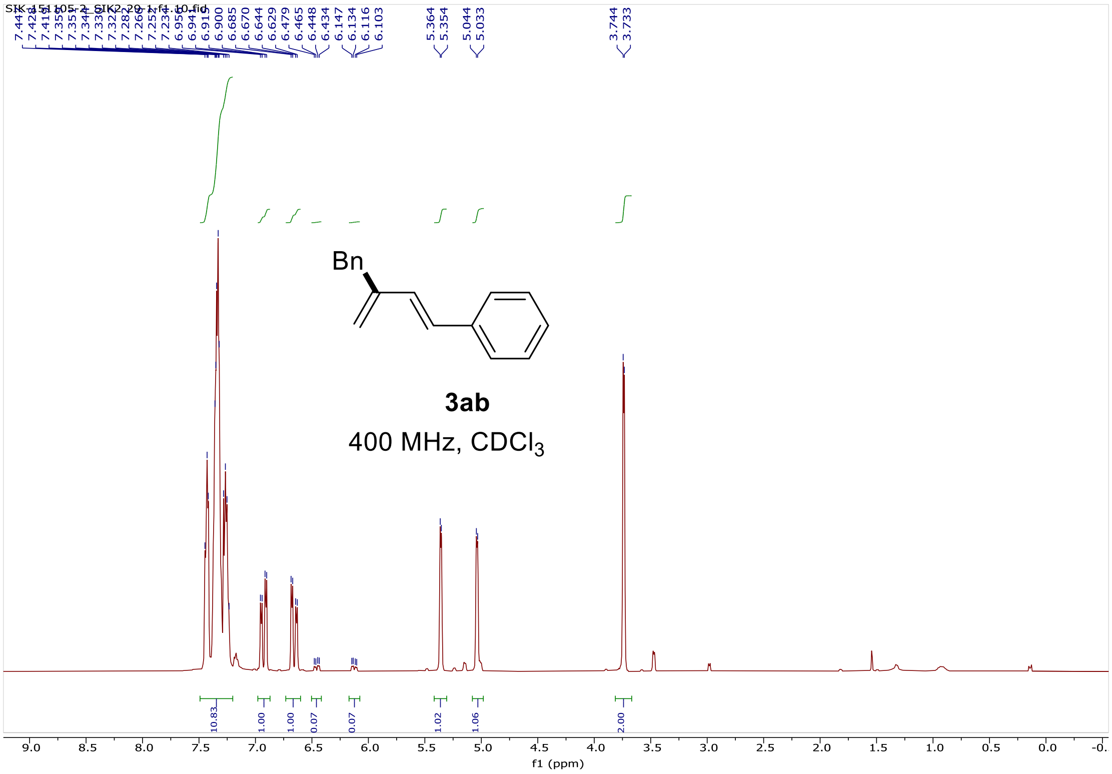
SIK-151105-2_SIK2-29-1-f1.10.fid
Bn
3ab
400 MHz, CDCl3
f1 (ppm)

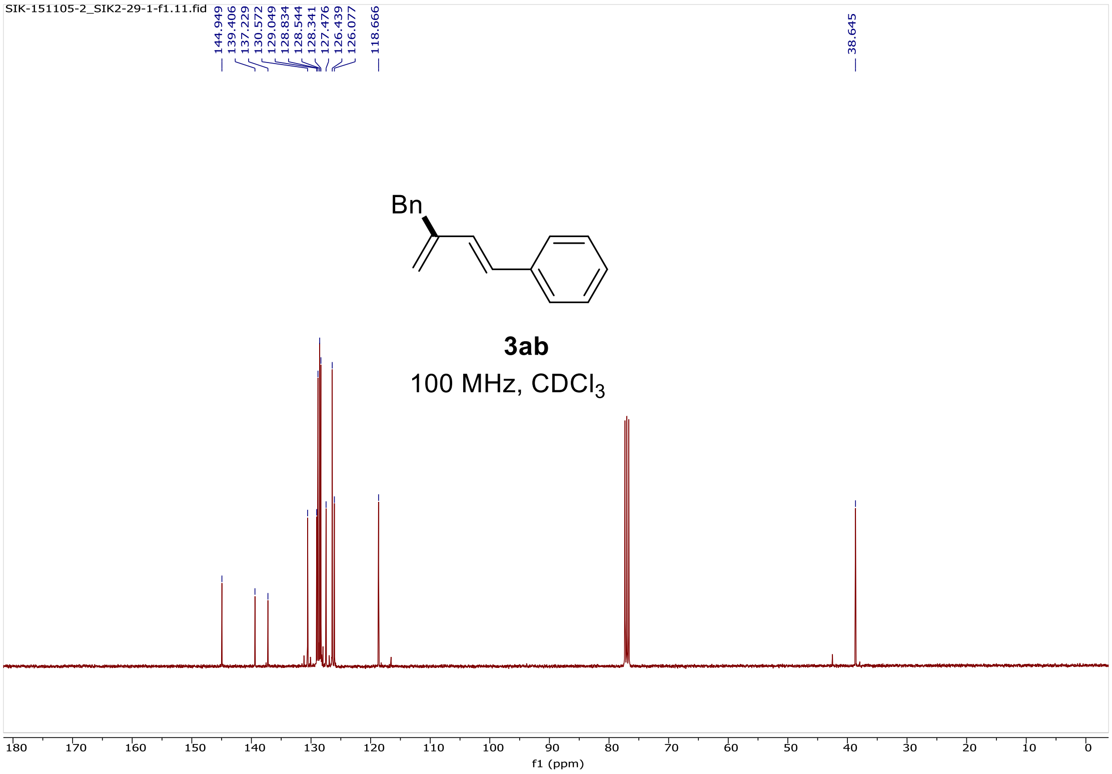
SIK-151105-2_SIK2-29-1-f1.11.fid
Bn
3ab
100 MHz, CDCl3
f1 (ppm)

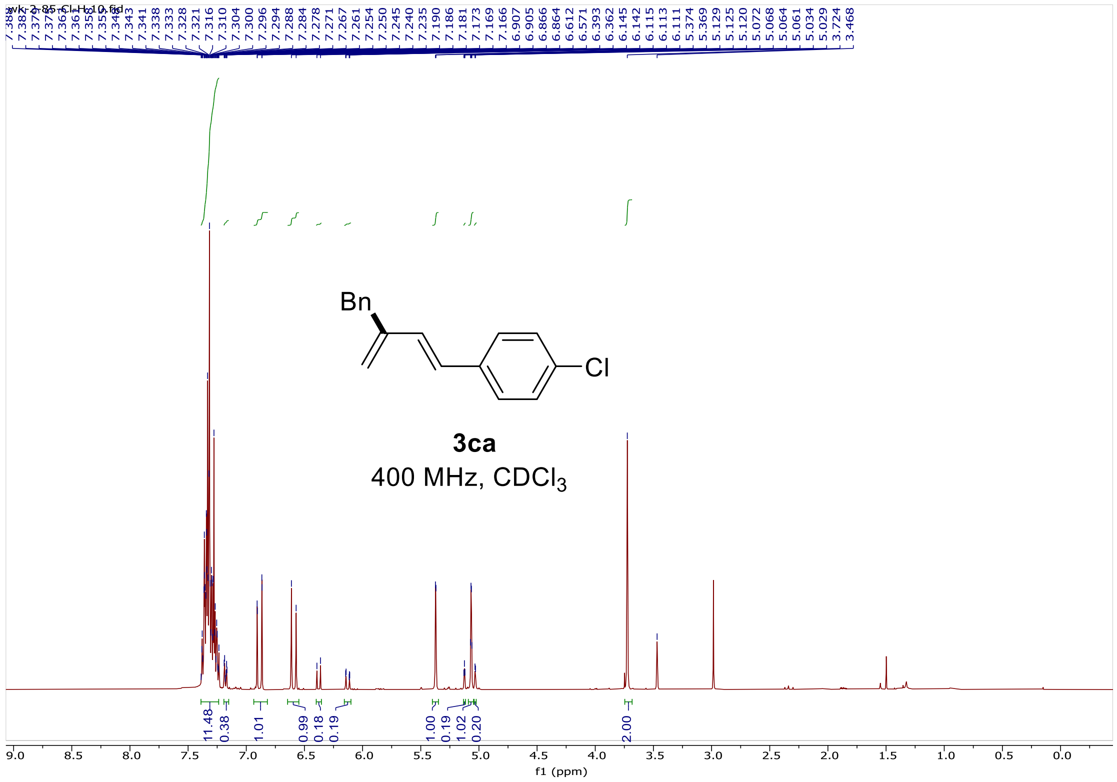

wk-2-85-Cl-H.10.fid
Bn
Cl
3ca
400 MHz, CDCl3
f1 (ppm)

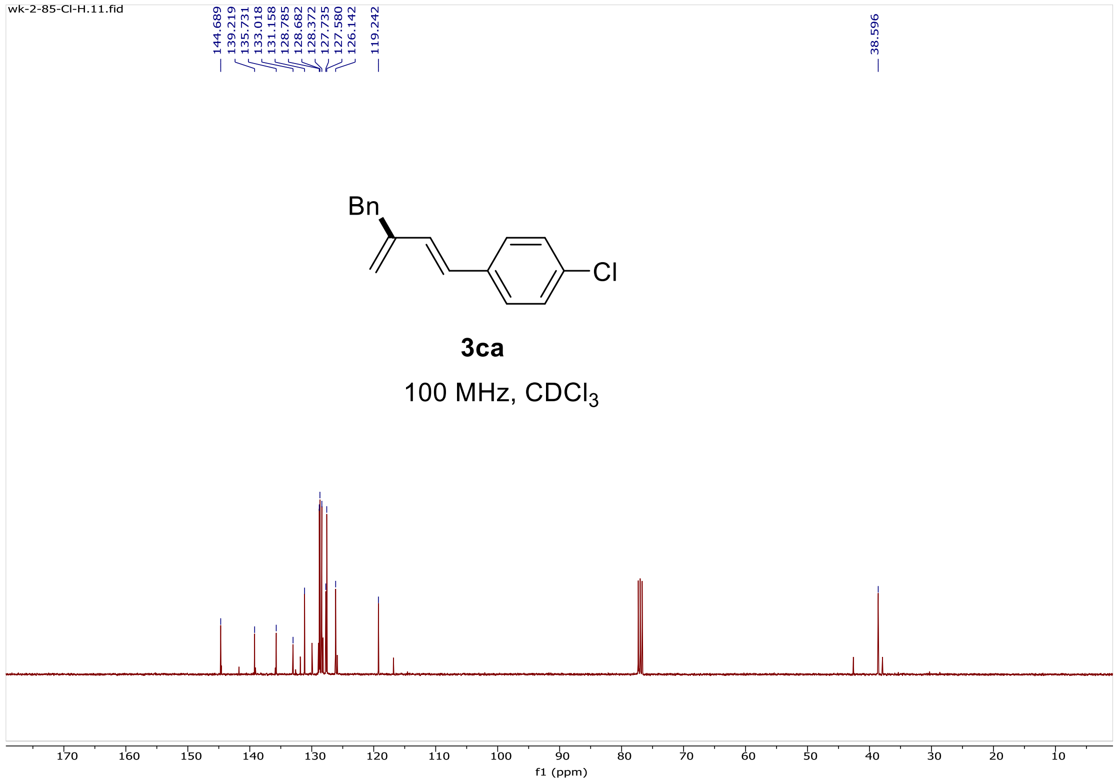

wk-2-85-Cl-H.11.fid
144.689
139.219
135.731
133.018
131.158
128.785
128.682
128.372
127.735
127.580
126.142
119.242
38.596
Bn
Cl
3ca
100 MHz, CDCl3
f1 (ppm)

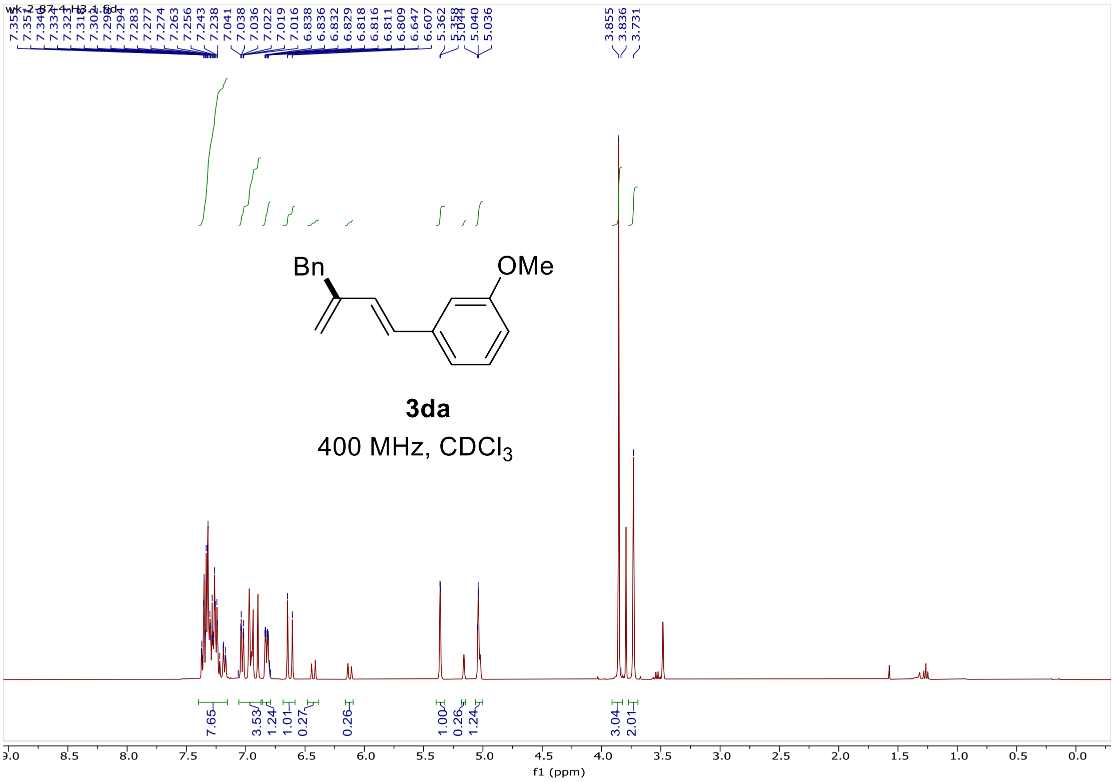
wk-2-87-4-H3.1.fid
Bn
OMe
3da
400 MHz, $CDCl_3$
f1 (ppm)

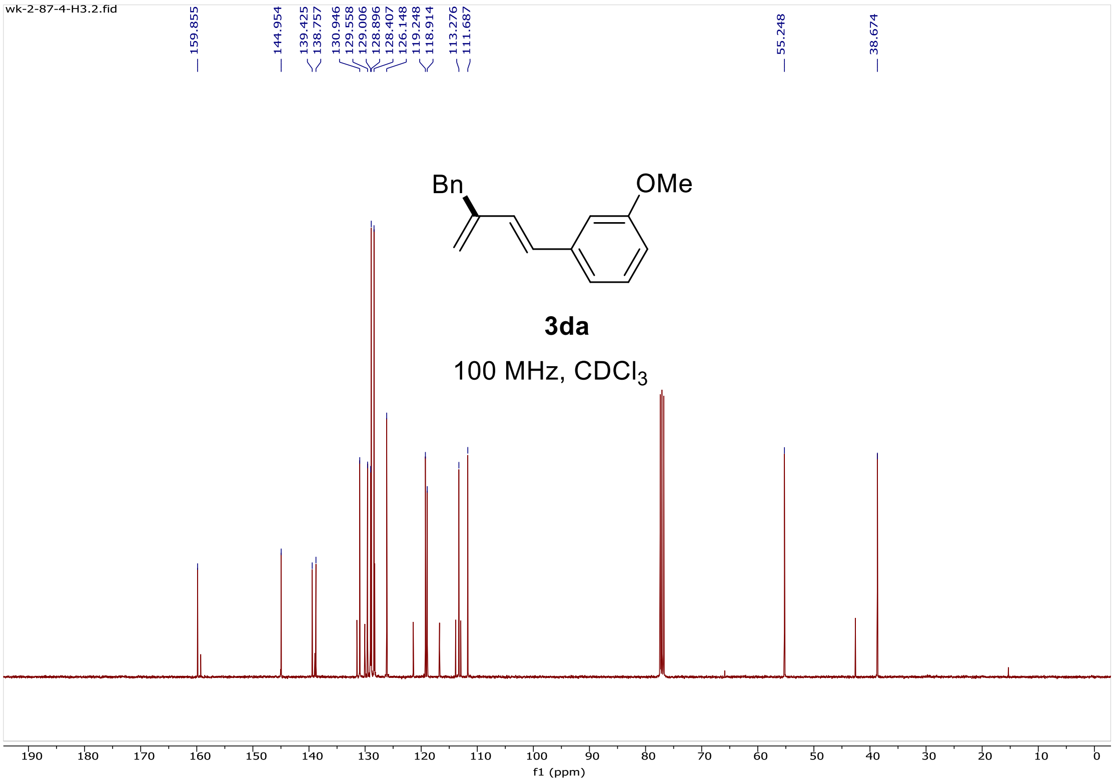
wk-2-87-4-H3.2.fid
159.855
144.954
139.425
138.757
130.946
129.558
129.006
128.896
128.407
126.148
119.248
118.914
113.276
111.687
55.248
38.674
Bn
OMe
3da
100 MHz, $CDCl_3$
f1 (ppm)

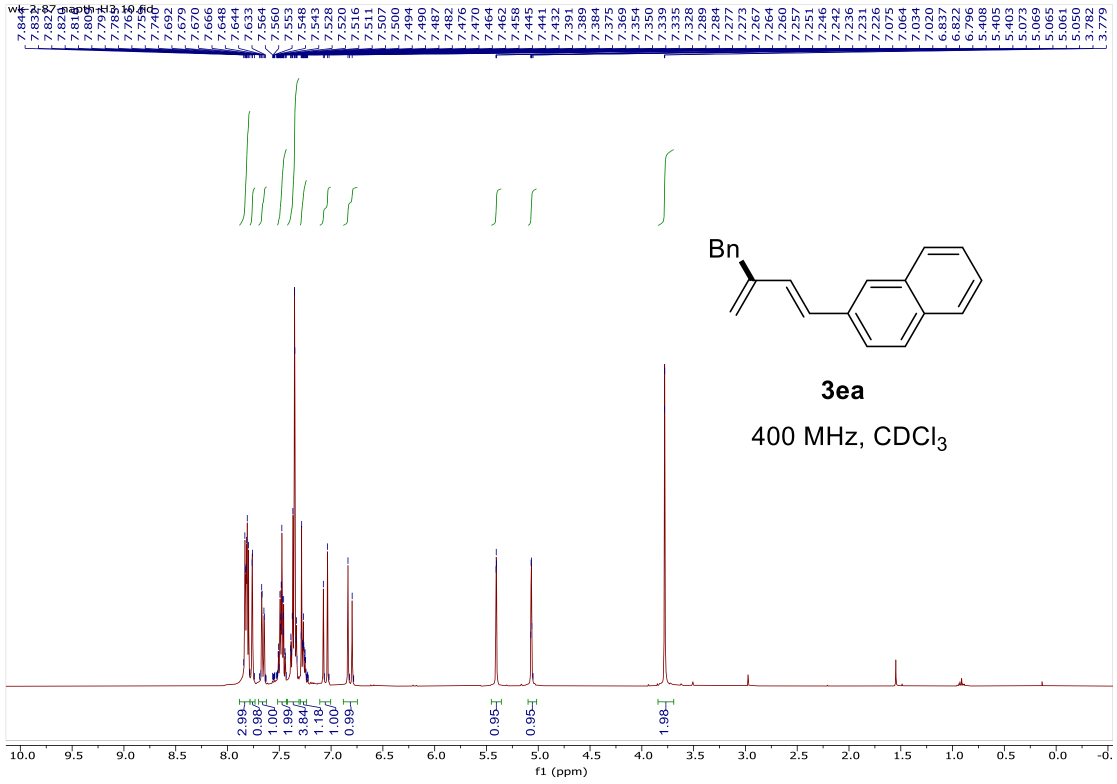
wk-2-87-napth-H3.10.fid
7.844 7.833 7.829 7.820 7.816 7.809 7.797 7.785 7.762 7.755 7.740 7.692 7.679 7.670 7.666 7.648 7.644 7.633 7.564 7.560 7.553 7.548 7.543 7.528 7.520 7.516 7.511 7.507 7.500 7.494 7.490 7.487 7.482 7.476 7.470 7.464 7.462 7.458 7.445 7.441 7.432 7.391 7.389 7.384 7.375 7.369 7.354 7.350 7.339 7.335 7.328 7.289 7.284 7.277 7.273 7.267 7.264 7.260 7.257 7.251 7.246 7.242 7.236 7.231 7.226 7.075 7.064 7.034 7.020 6.837 6.822 6.796 5.408 5.405 5.403 5.073 5.069 5.065 5.061 5.050 3.782 3.779
Bn
3ea
400 MHz, CDCl3
2.99 0.98 1.00 1.99 3.84 1.18 1.00 0.99 0.95 0.95 1.98
f1 (ppm)

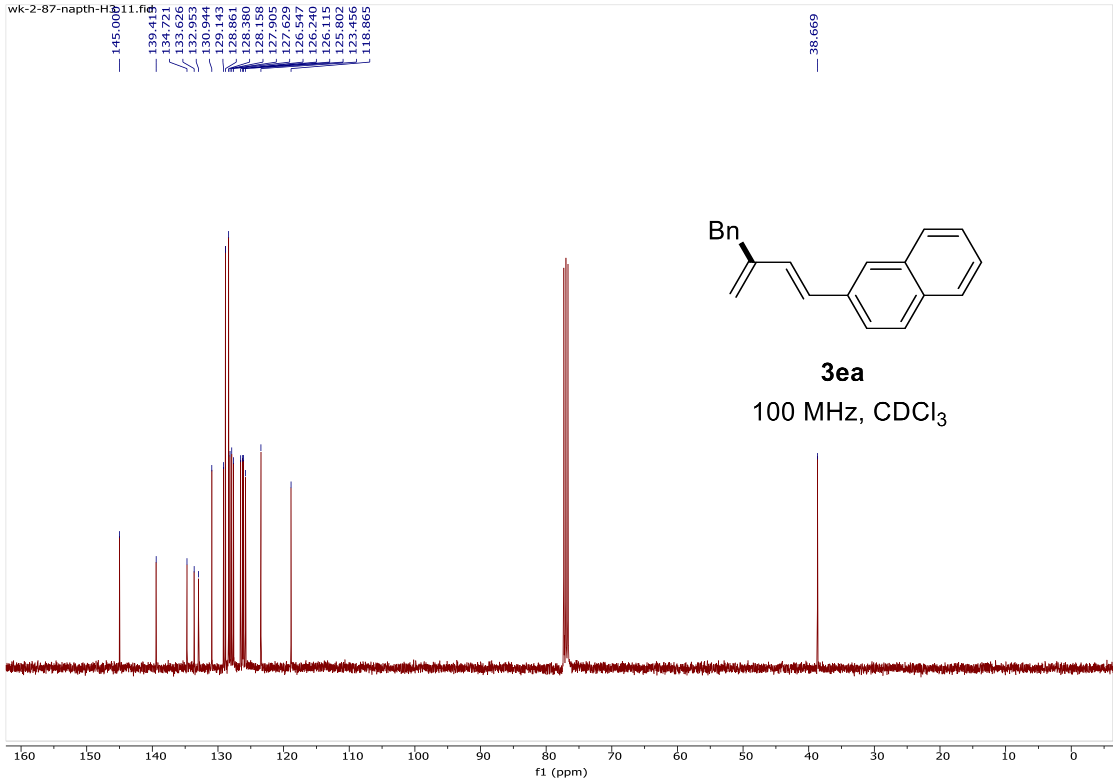
wk-2-87-napth-H3.11.fid
145.000 139.415 134.721 133.626 132.953 130.944 129.143 128.861 128.380 128.158 127.905 127.629 126.547 126.240 126.115 125.802 123.456 118.865 38.669
Bn
3ea
100 MHz, CDCl3
f1 (ppm)

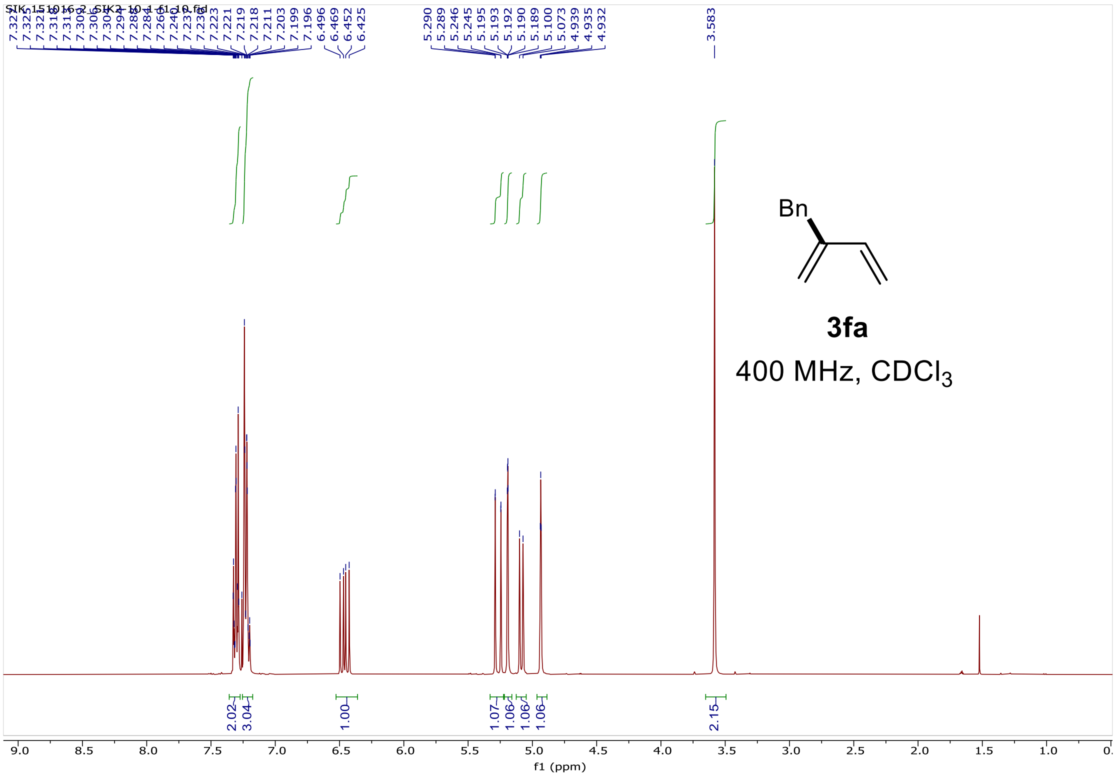
SIK-151016-2_SIK2-10-1-f1.10.fid
3fa
400 MHz, $CDCl_3$
f1 (ppm)

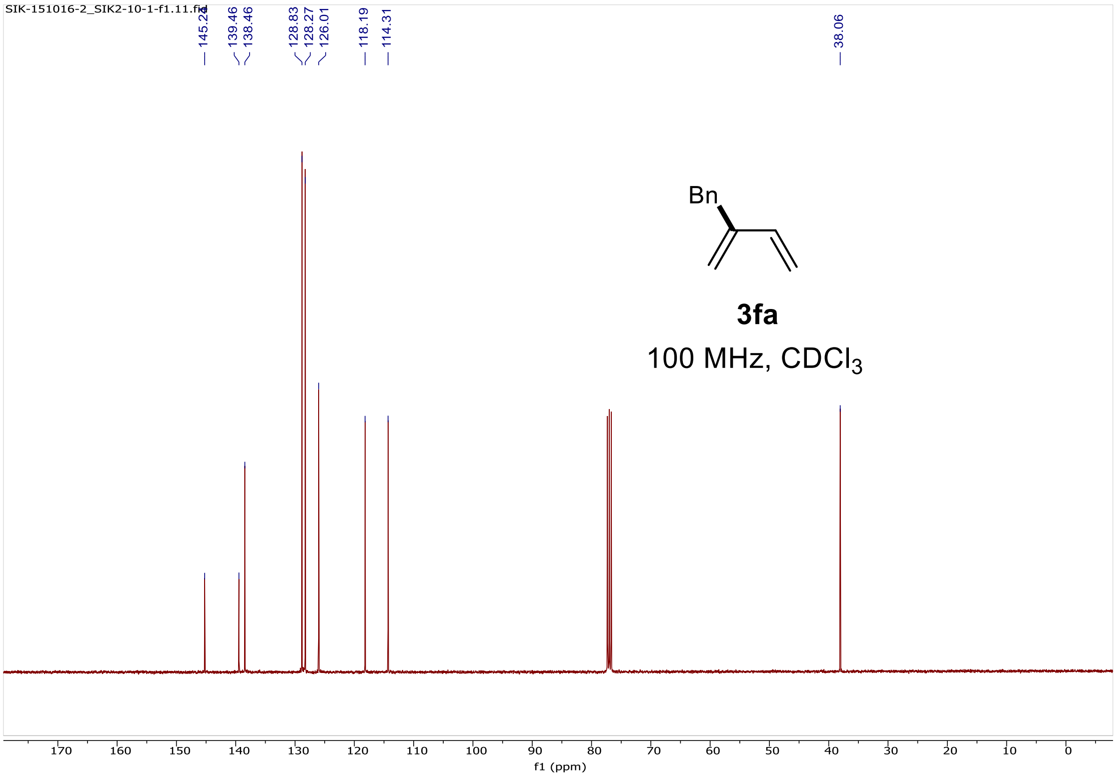
SIK-151016-2_SIK2-10-1-f1.11.fid
3fa
100 MHz, $CDCl_3$
f1 (ppm)

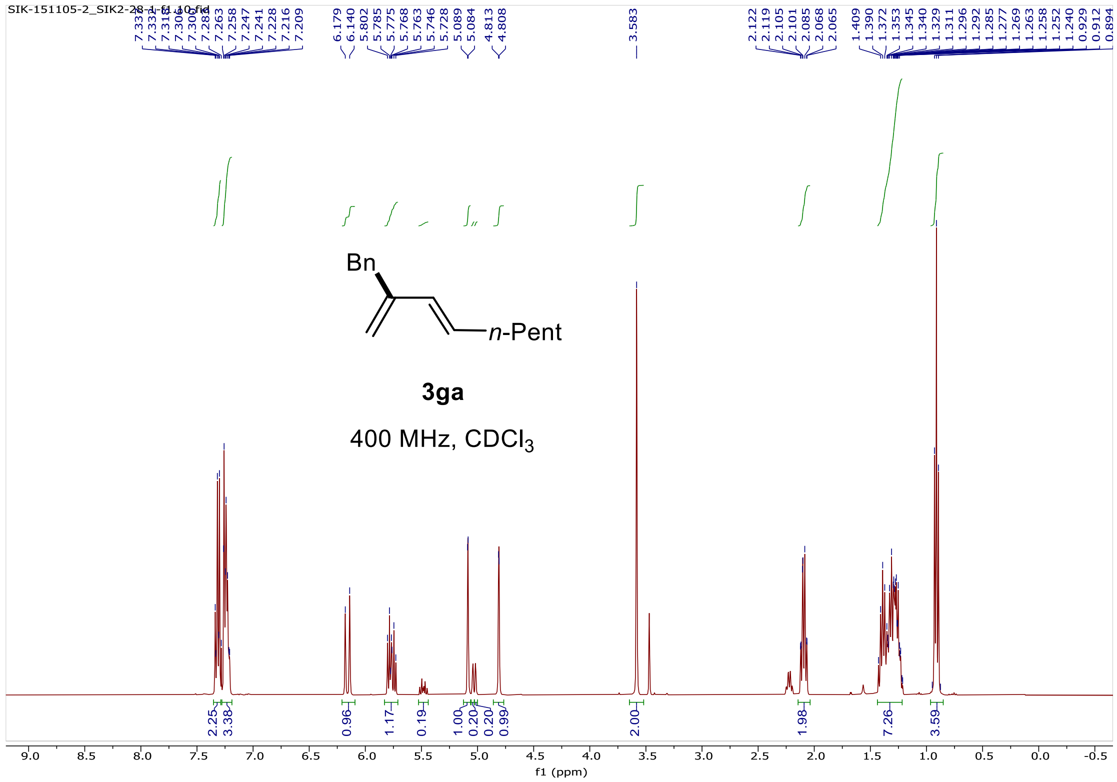
SIK-151105-2_SIK2-28-1-f1.10.fid
Bn
n-Pent
3ga
400 MHz, CDCl3
f1 (ppm)

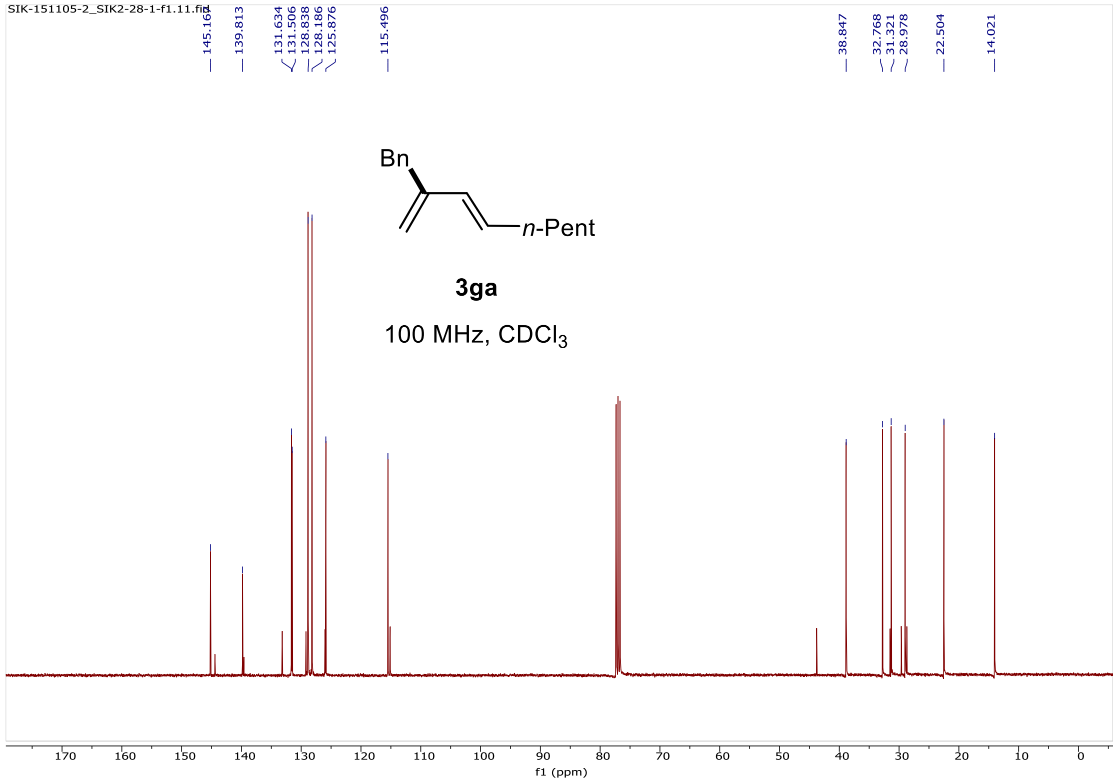
SIK-151105-2_SIK2-28-1-f1.11.fid
145.161
139.813
131.634
131.506
128.838
128.186
125.876
115.496
38.847
32.768
31.321
28.978
22.504
14.021
Bn
n-Pent
3ga
100 MHz, CDCl3
f1 (ppm)

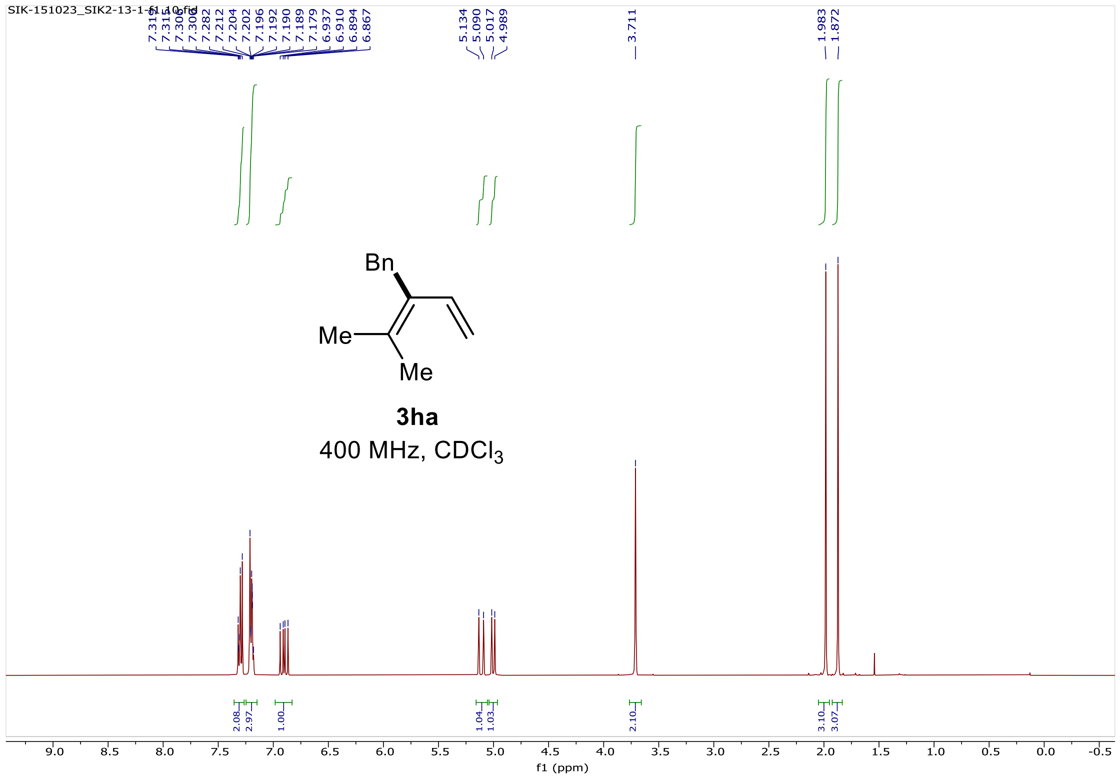
SIK-151023_SIK2-13-1-f1.10.fid
3ha
400 MHz, CDCl3
f1 (ppm)

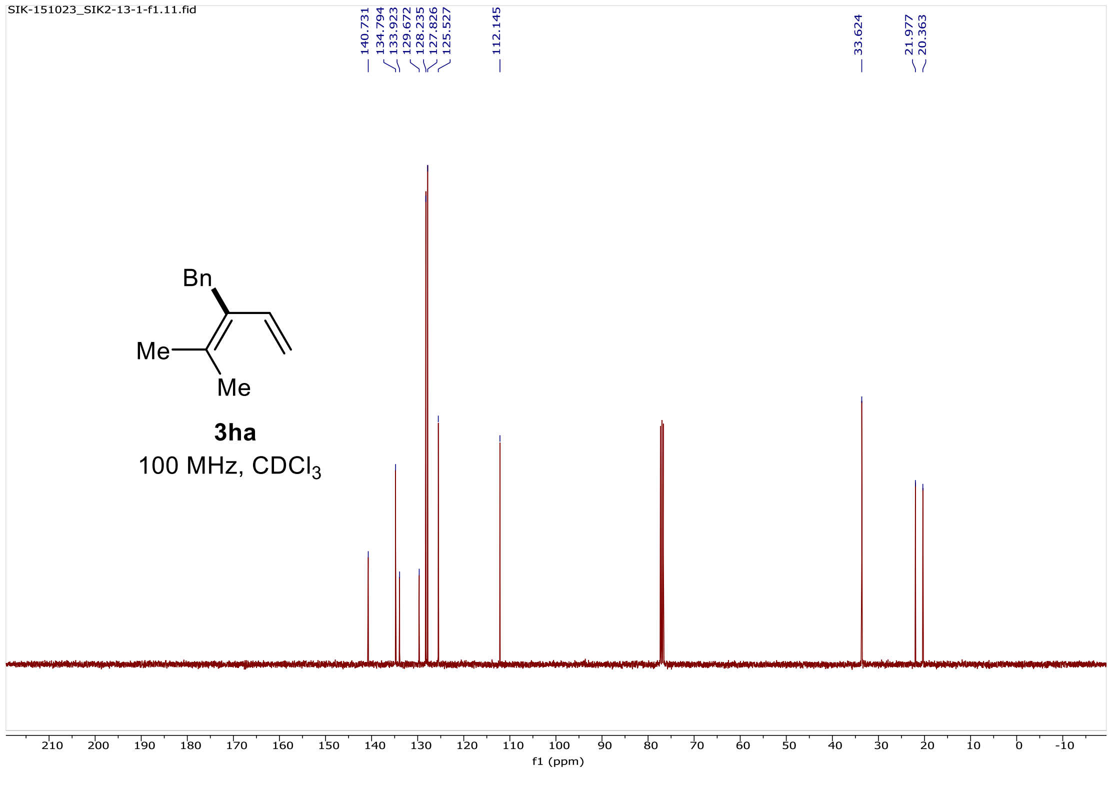
SIK-151023_SIK2-13-1-f1.11.fid
3ha
100 MHz, CDCl3
f1 (ppm)

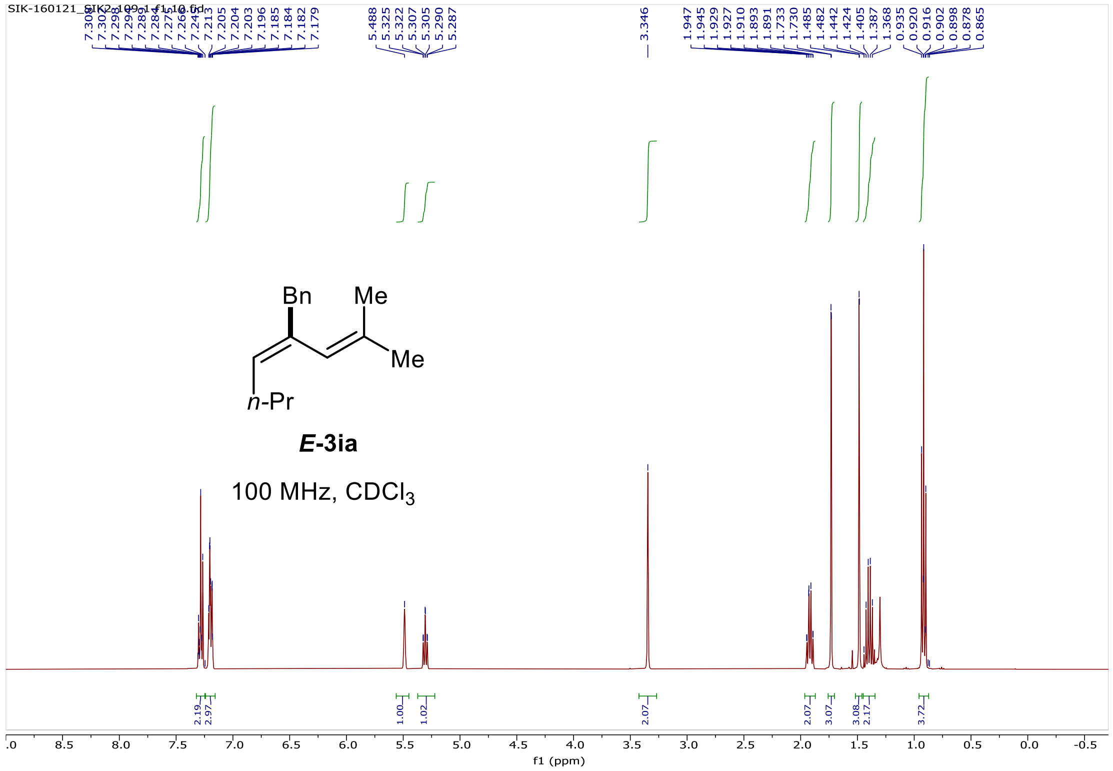
SIK-160121_SIK2-109-1-f1.10.fid
5.488
5.325
5.322
5.307
5.305
5.290
5.287
3.346
1.947
1.945
1.929
1.927
1.910
1.893
1.891
1.733
1.730
1.485
1.482
1.442
1.424
1.405
1.387
1.368
0.935
0.920
0.916
0.902
0.898
0.878
0.865
Bn
Me
Me
n-Pr
E-3ia
100 MHz, CDCl3
2.19
2.97
1.00
1.02
2.07
2.07
3.07
3.08
2.17
3.72
f1 (ppm)

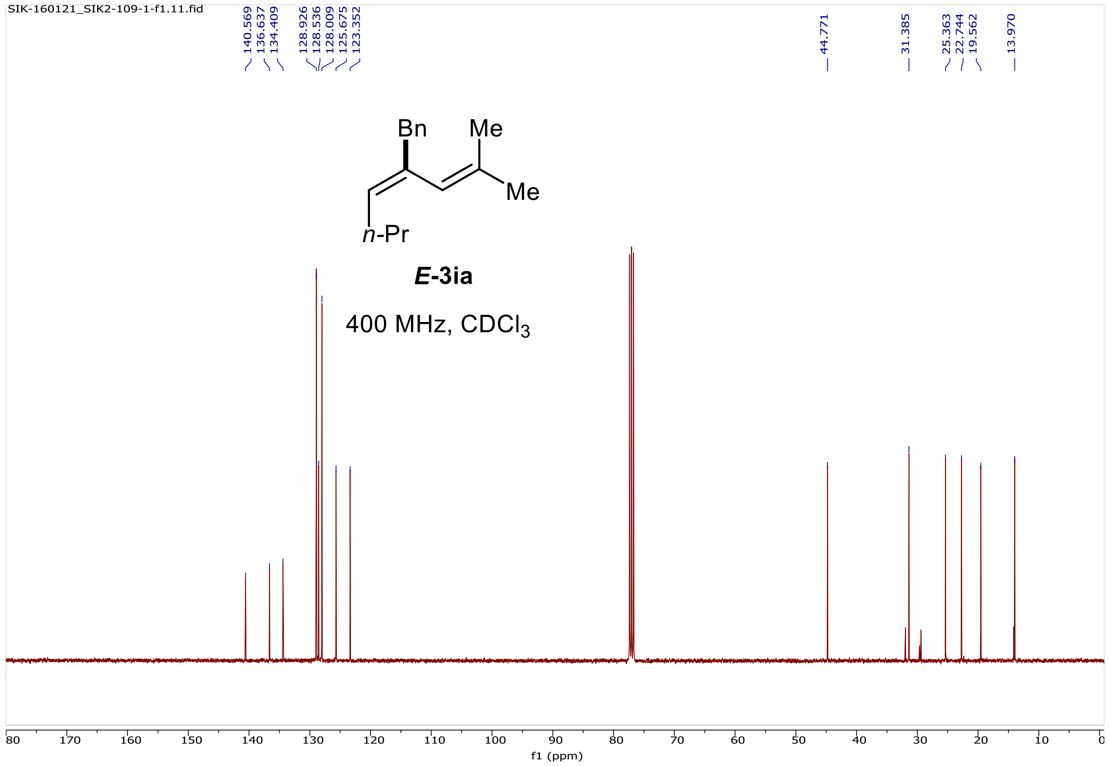
SIK-160121_SIK2-109-1-f1.11.fid
140.569
136.637
134.409
128.926
128.536
128.009
125.675
123.352
44.771
31.385
25.363
22.744
19.562
13.970
Bn
Me
Me
n-Pr
E-3ia
400 MHz, CDCl3
f1 (ppm)

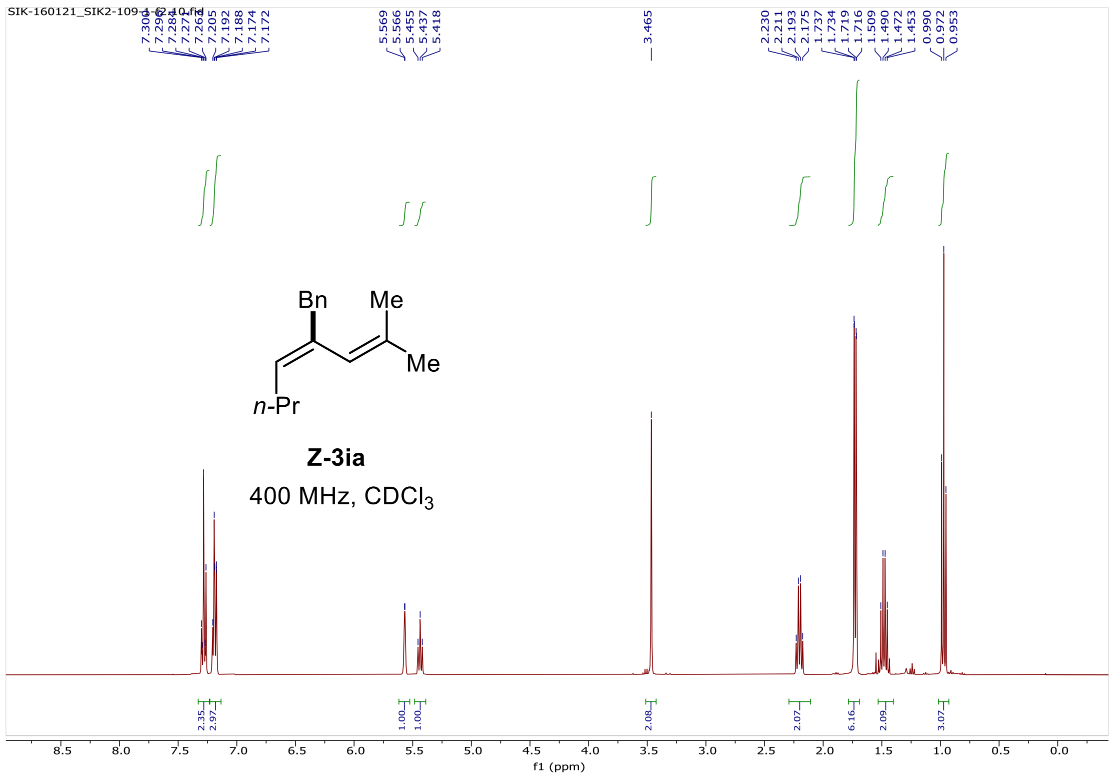
SIK-160121_SIK2-109-1-f2.10.fid
Bn
Me
Me
n-Pr
Z-3ia
400 MHz, CDCl3
f1 (ppm)

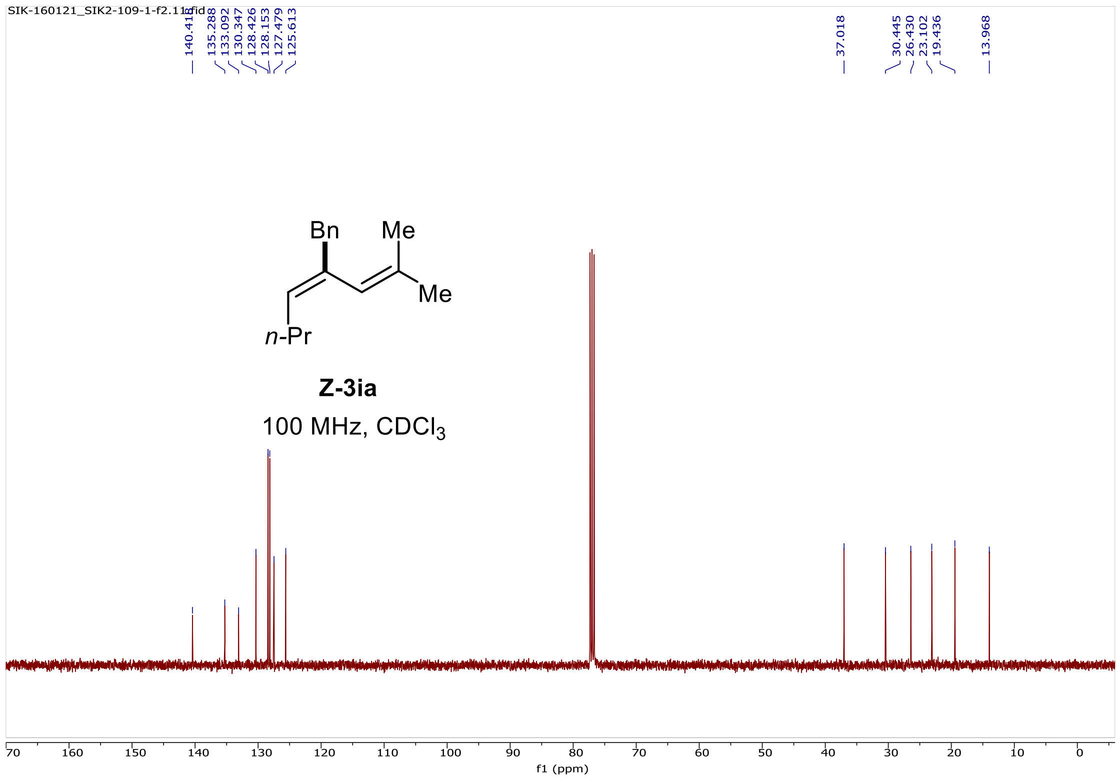
SIK-160121_SIK2-109-1-f2.11.fid
140.415
135.288
133.092
130.347
128.426
128.153
127.479
125.613
37.018
30.445
26.430
23.102
19.436
13.968
Bn
Me
Me
n-Pr
Z-3ia
100 MHz, CDCl3
f1 (ppm)

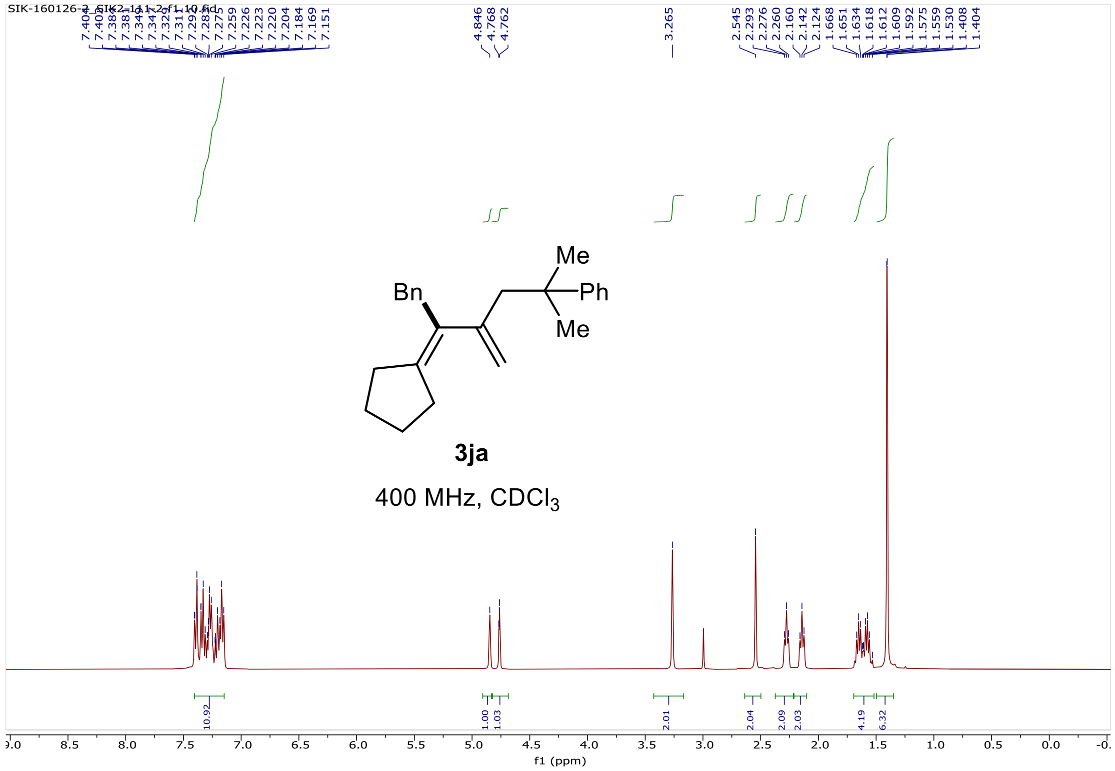
SIK-160126-2_SIK2-111-2-f1.10.fid
3ja
400 MHz, CDCl3
f1 (ppm)

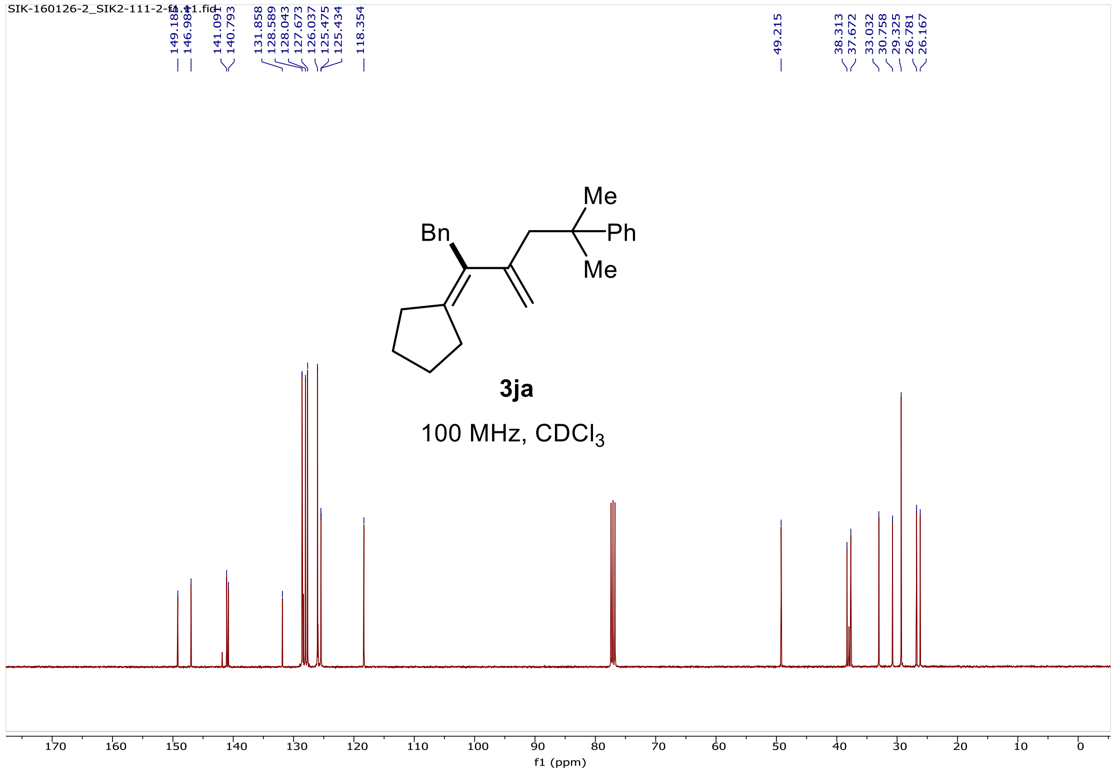
SIK-160126-2_SIK2-111-2-f1.11.fid
3ja
100 MHz, CDCl3
f1 (ppm)

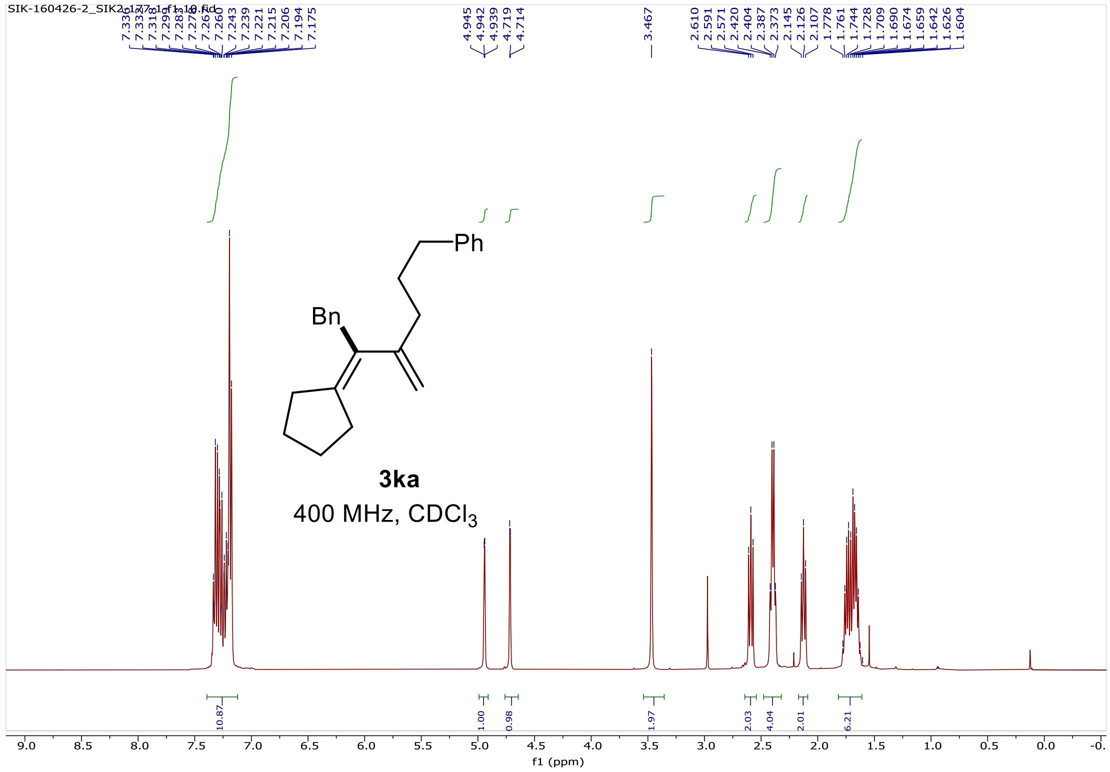

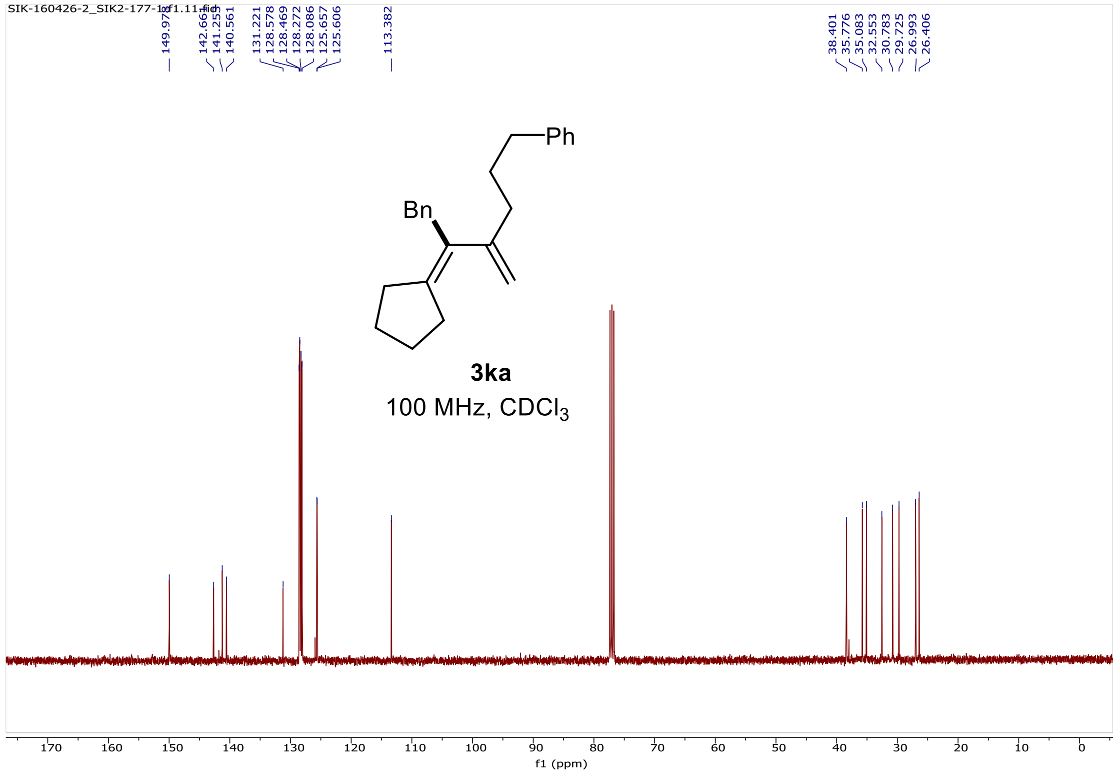

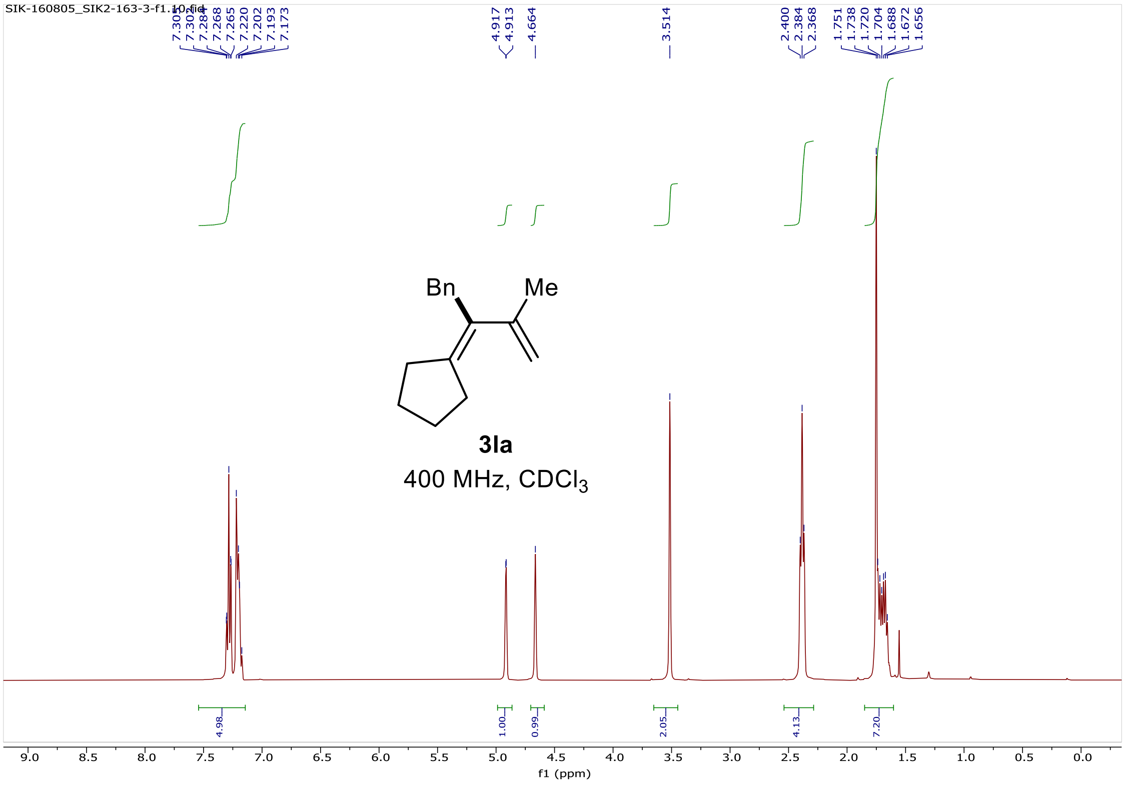
SIK-160805_SIK2-163-3-f1.10.fid
7.303
7.300
7.284
7.268
7.265
7.220
7.202
7.193
7.173
4.917
4.913
4.664
3.514
2.400
2.384
2.368
1.751
1.738
1.720
1.704
1.688
1.672
1.656
Bn
Me
3la
400 MHz, CDCl3
4.98
1.00
0.99
2.05
4.13
7.20
f1 (ppm)

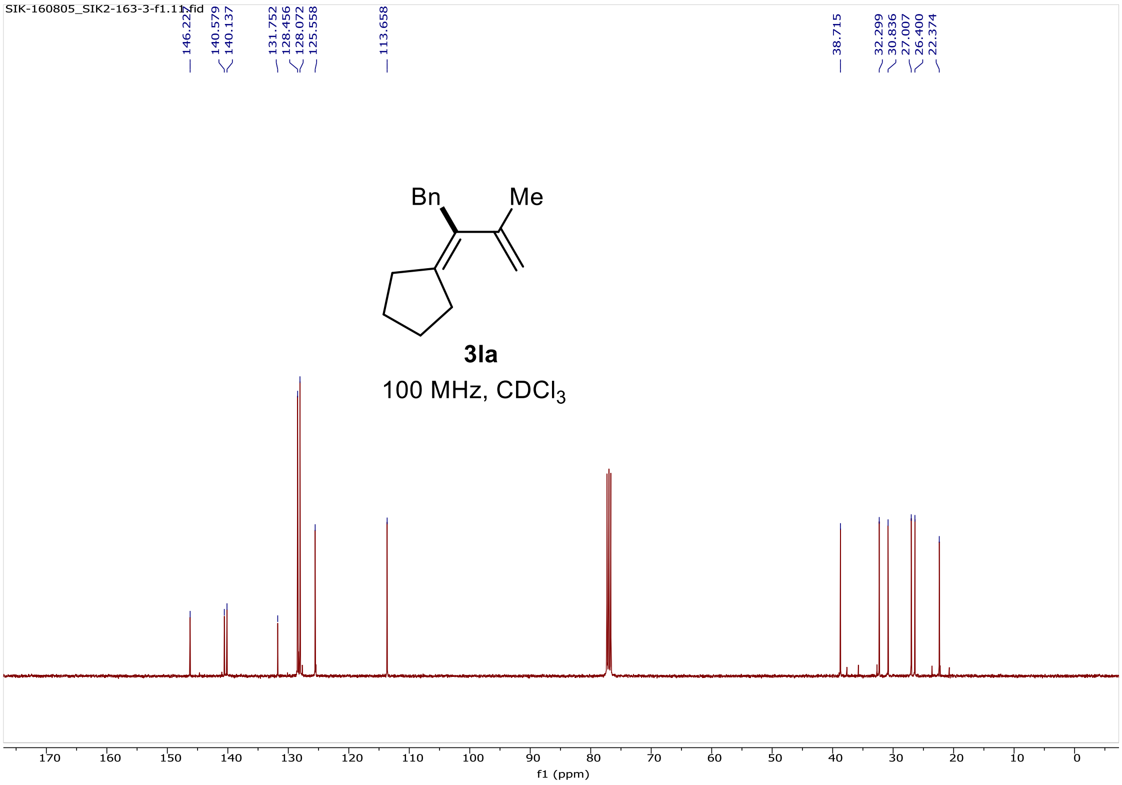
SIK-160805_SIK2-163-3-f1.11.fid
146.222
140.579
140.137
131.752
128.456
128.072
125.558
113.658
38.715
32.299
30.836
27.007
26.400
22.374
Bn
Me
3la
100 MHz, CDCl3
f1 (ppm)

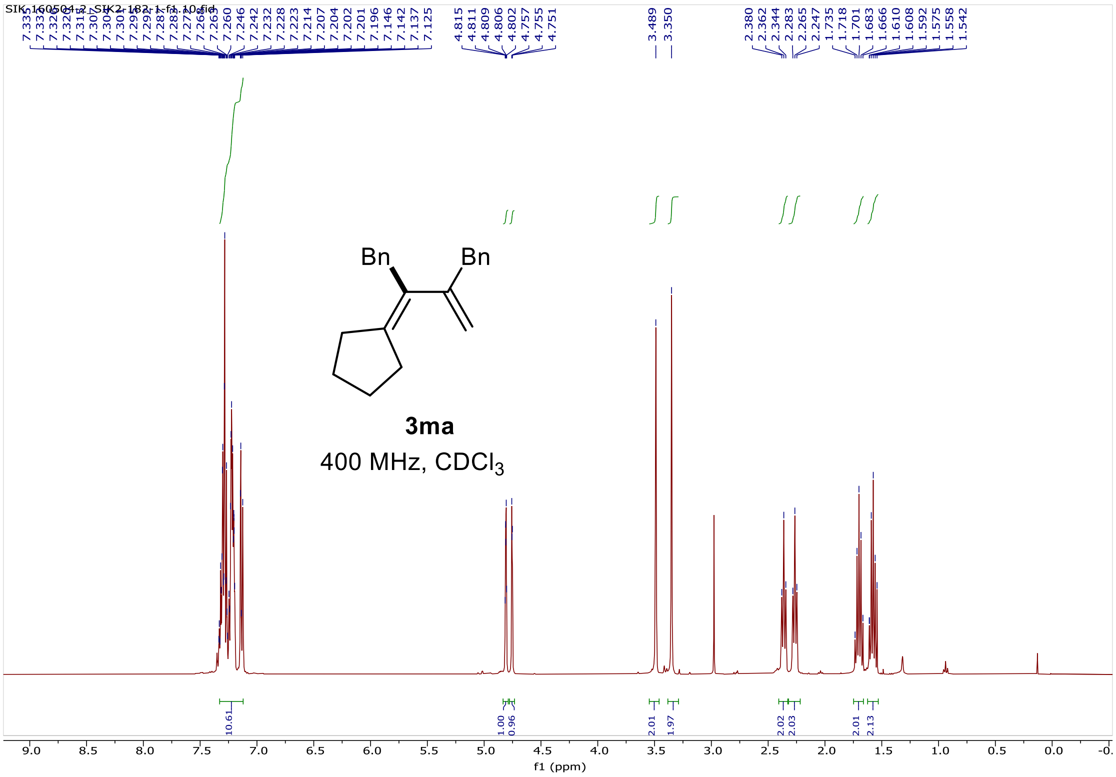
SIK-160504-2_SIK2-182-1-f1.10.fid
7.263
7.260
7.246
7.242
7.232
7.228
7.223
7.214
7.207
7.204
7.202
7.201
7.196
7.146
7.142
7.137
7.125
4.815
4.811
4.809
4.806
4.802
4.757
4.755
4.751
3.489
3.350
2.380
2.362
2.344
2.283
2.265
2.247
1.735
1.718
1.701
1.683
1.666
1.610
1.608
1.592
1.575
1.558
1.542
Bn
Bn
3ma
400 MHz, CDCl3
10.61
1.00
0.96
2.01
1.97
2.02
2.03
2.01
2.13
f1 (ppm)

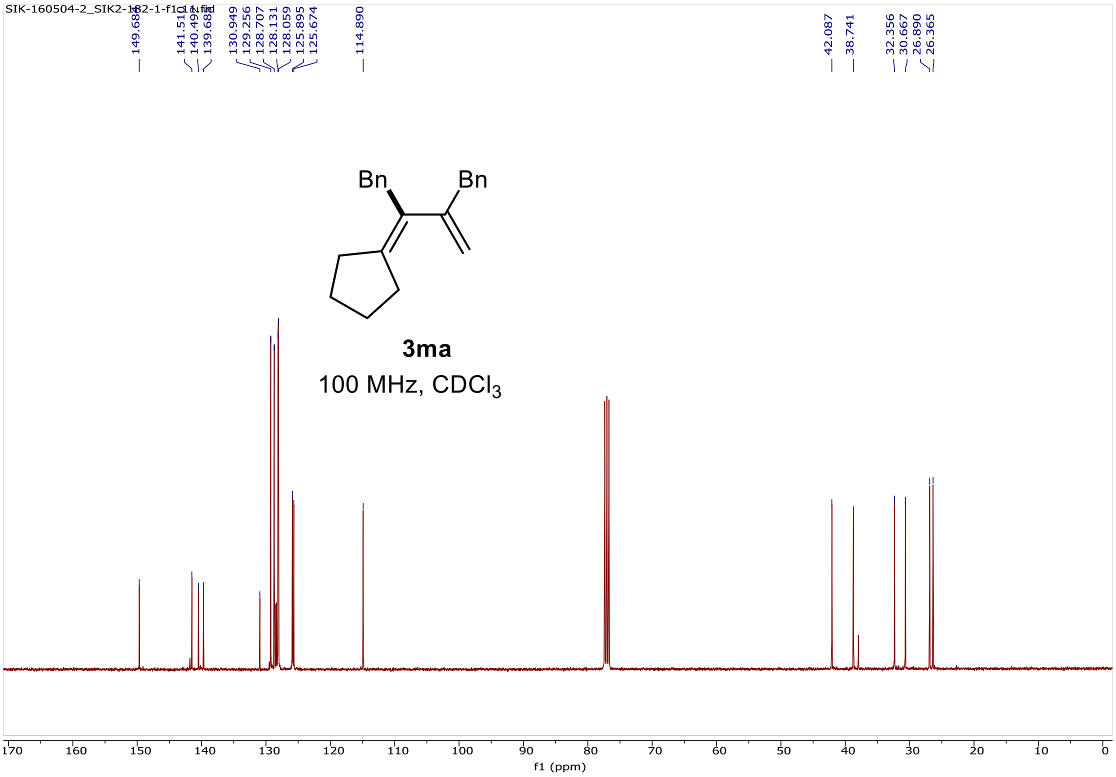
SIK-160504-2_SIK2-182-1-f1C.1.fid
149.688
141.510
140.496
139.688
130.949
129.256
128.707
128.131
128.059
125.895
125.674
114.890
42.087
38.741
32.356
30.667
26.890
26.365
Bn
Bn
3ma
100 MHz, CDCl3
f1 (ppm)

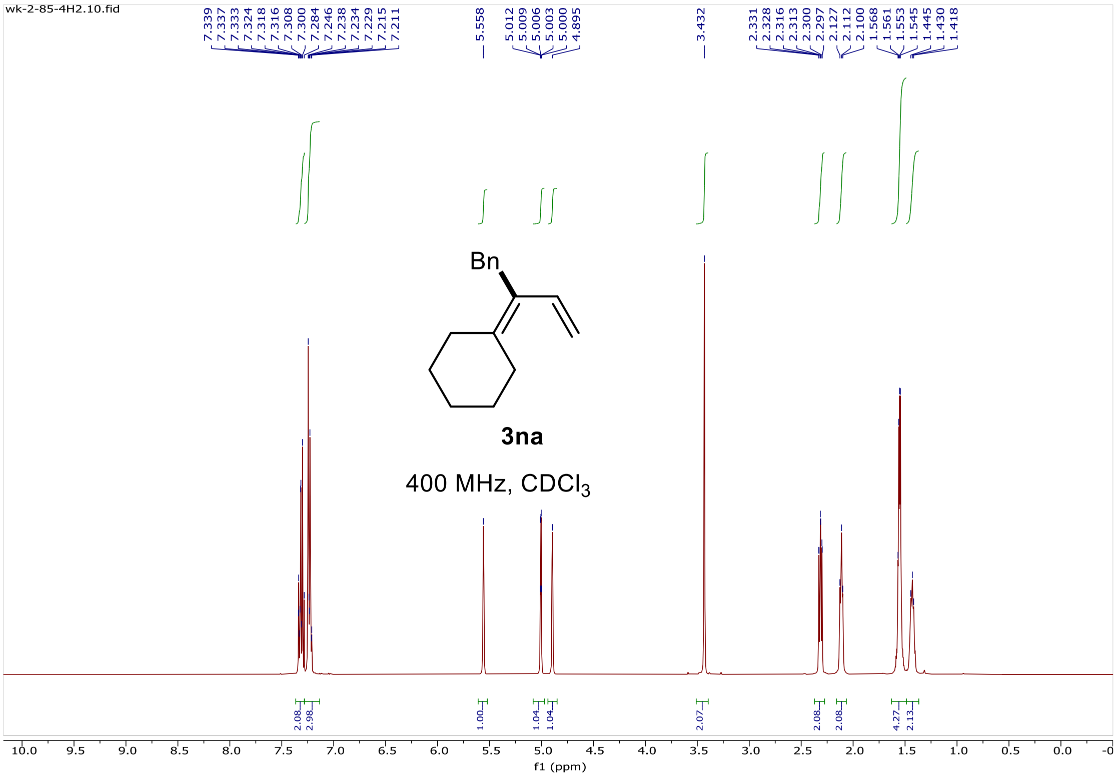
wk-2-85-4H2.10.fid
7.339 7.337 7.333 7.324 7.318 7.316 7.308 7.300 7.284 7.246 7.238 7.234 7.229 7.215 7.211
5.558
5.012 5.009 5.006 5.003 5.000 4.895
3.432
2.331 2.328 2.316 2.313 2.300 2.297 2.127 2.112 2.100
1.568 1.561 1.553 1.545 1.445 1.430 1.418
Bn
3na
400 MHz, CDCl3
2.08 2.98 1.00 1.04 1.04 2.07 2.08 2.08 4.27 2.13
f1 (ppm)

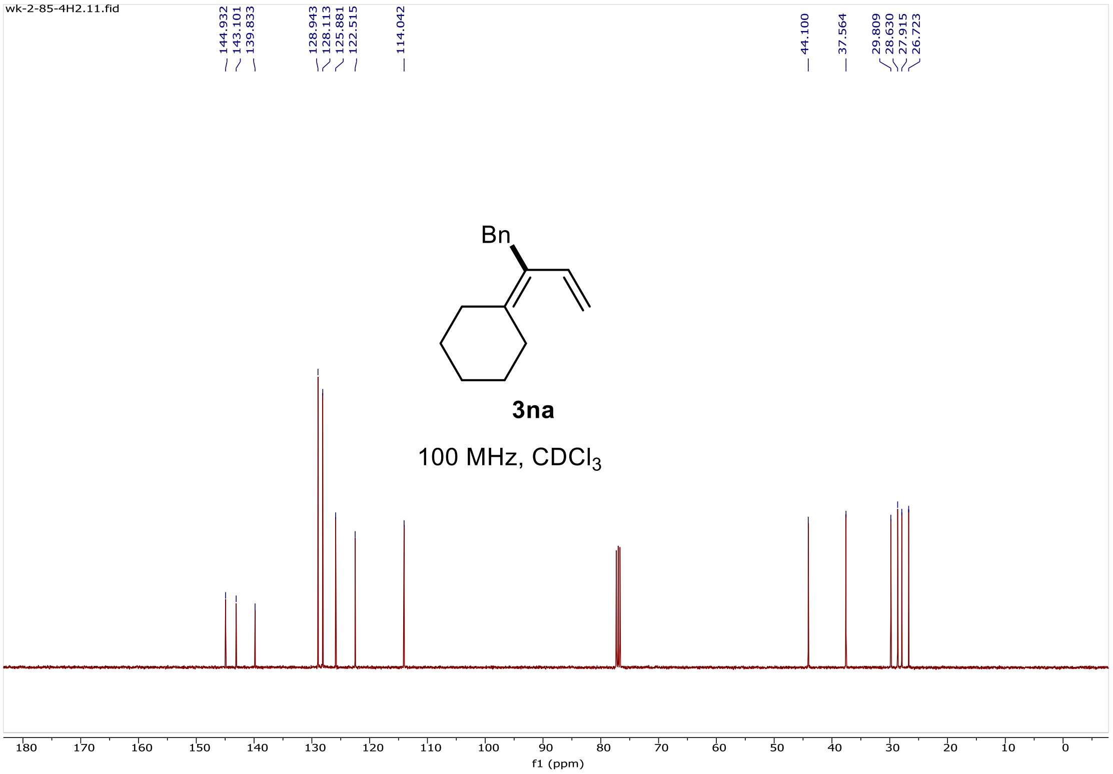
wk-2-85-4H2.11.fid
144.932 143.101 139.833
128.943 128.113 125.881 122.515
114.042
44.100
37.564
29.809 28.630 27.915 26.723
Bn
3na
100 MHz, CDCl3
f1 (ppm)

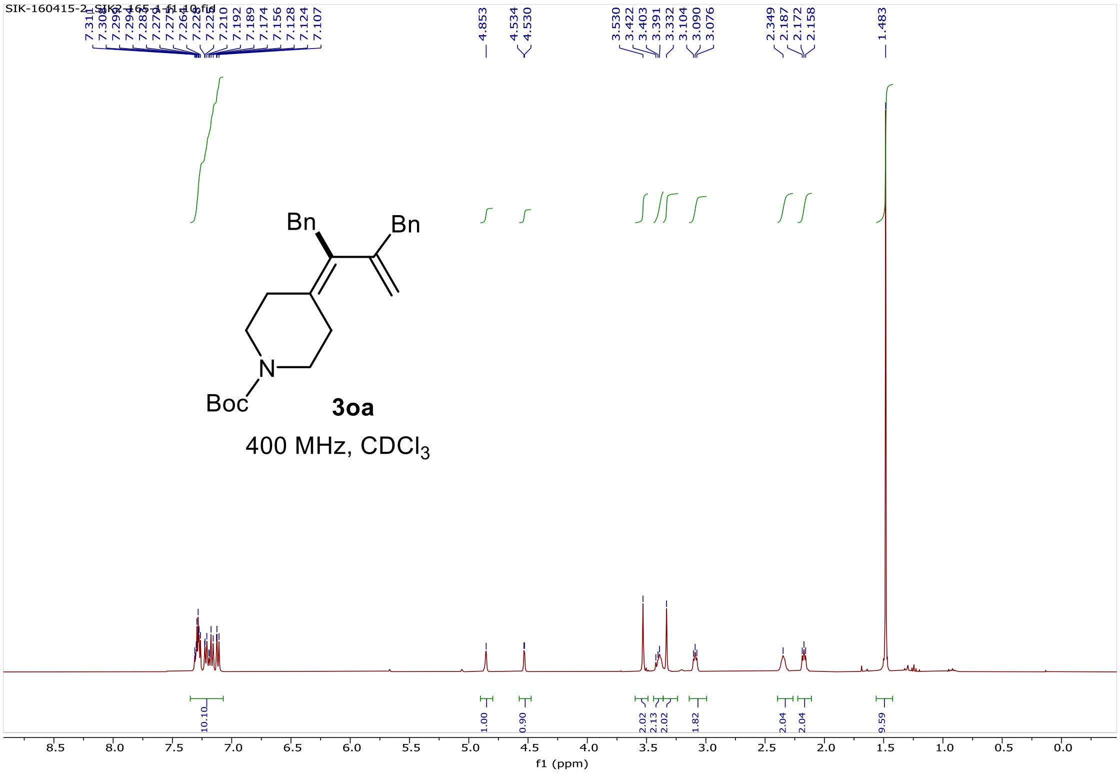
SIK-160415-2_SIK2-165-1-f1.10.fid
Bn
Bn
N
Boc
3oa
400 MHz, CDCl3
f1 (ppm)

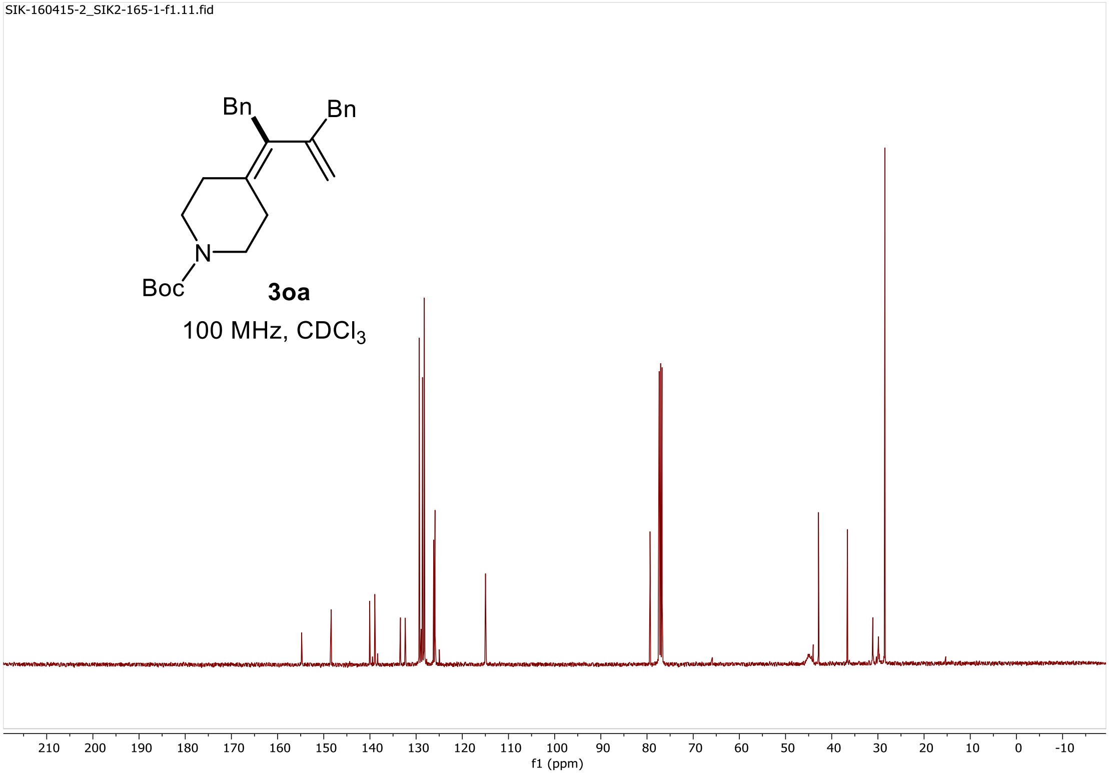
SIK-160415-2_SIK2-165-1-f1.11.fid
Bn
Bn
N
Boc
3oa
100 MHz, CDCl3
f1 (ppm)

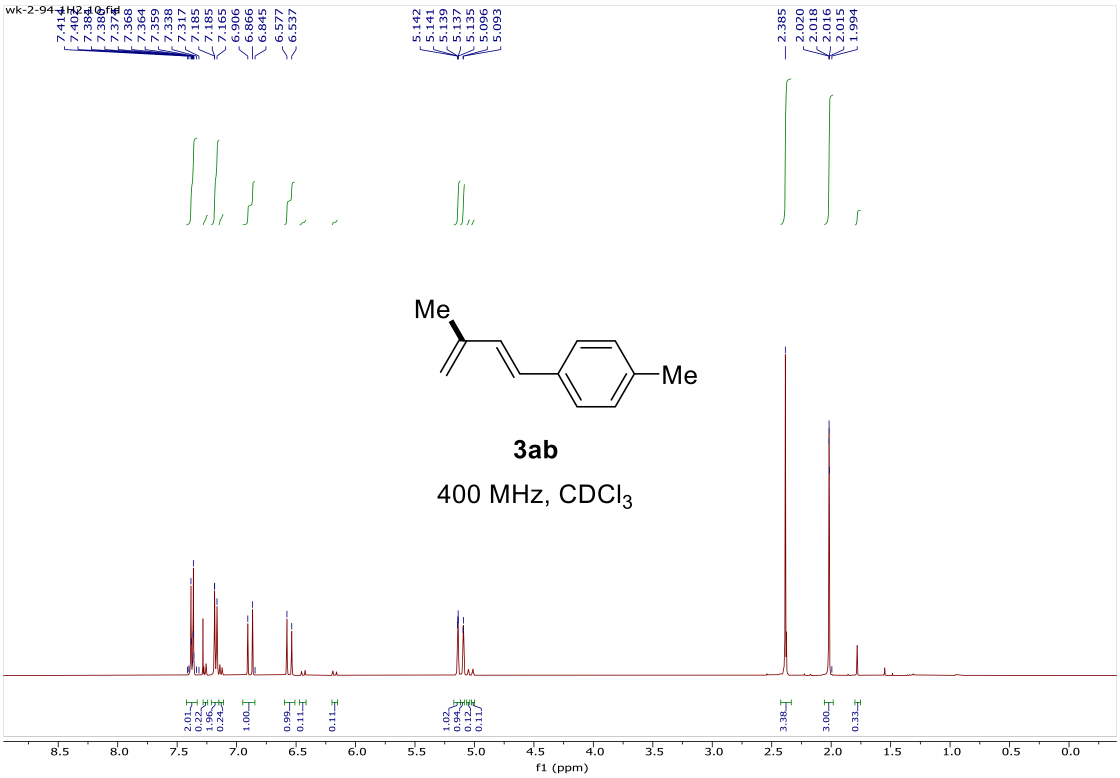
wk-2-94-1H2.10.fid
3ab
400 MHz, CDCl3
f1 (ppm)

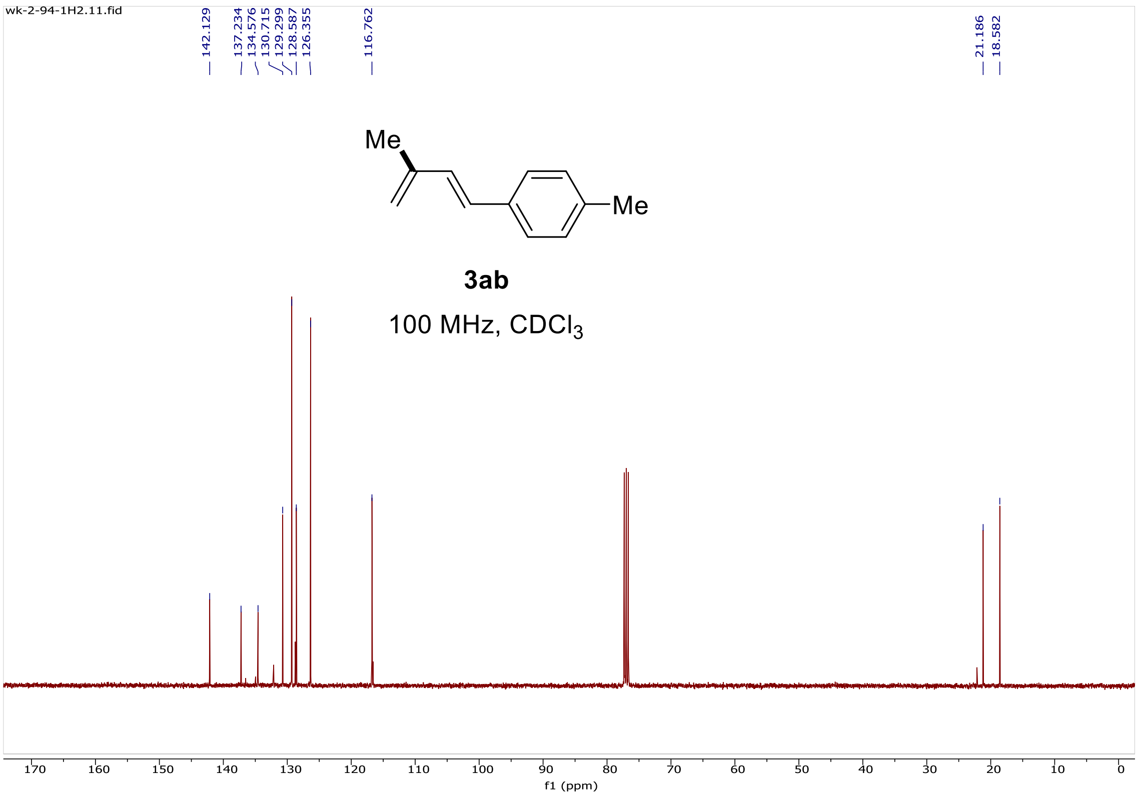
wk-2-94-1H2.11.fid
142.129
137.234
134.576
130.715
129.299
128.587
126.355
116.762
21.186
18.582
3ab
100 MHz, CDCl3
f1 (ppm)

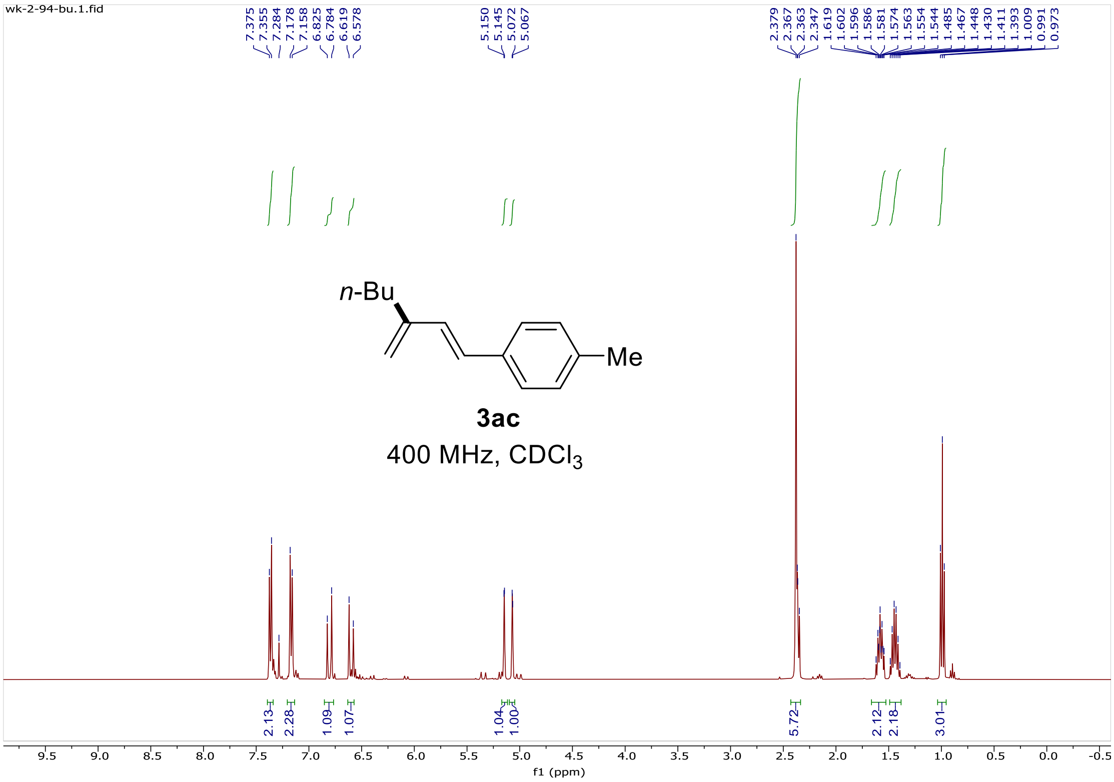
wk-2-94-bu.1.fid
7.375
7.355
7.284
7.178
7.158
6.825
6.784
6.619
6.578
5.150
5.145
5.072
5.067
2.379
2.367
2.363
2.347
1.619
1.602
1.596
1.586
1.581
1.574
1.563
1.554
1.544
1.485
1.467
1.448
1.430
1.411
1.393
1.009
0.991
0.973
n-Bu
Me
3ac
400 MHz, CDCl3
2.13
2.28
1.09
1.07
1.04
1.00
5.72
2.12
2.18
3.01
f1 (ppm)

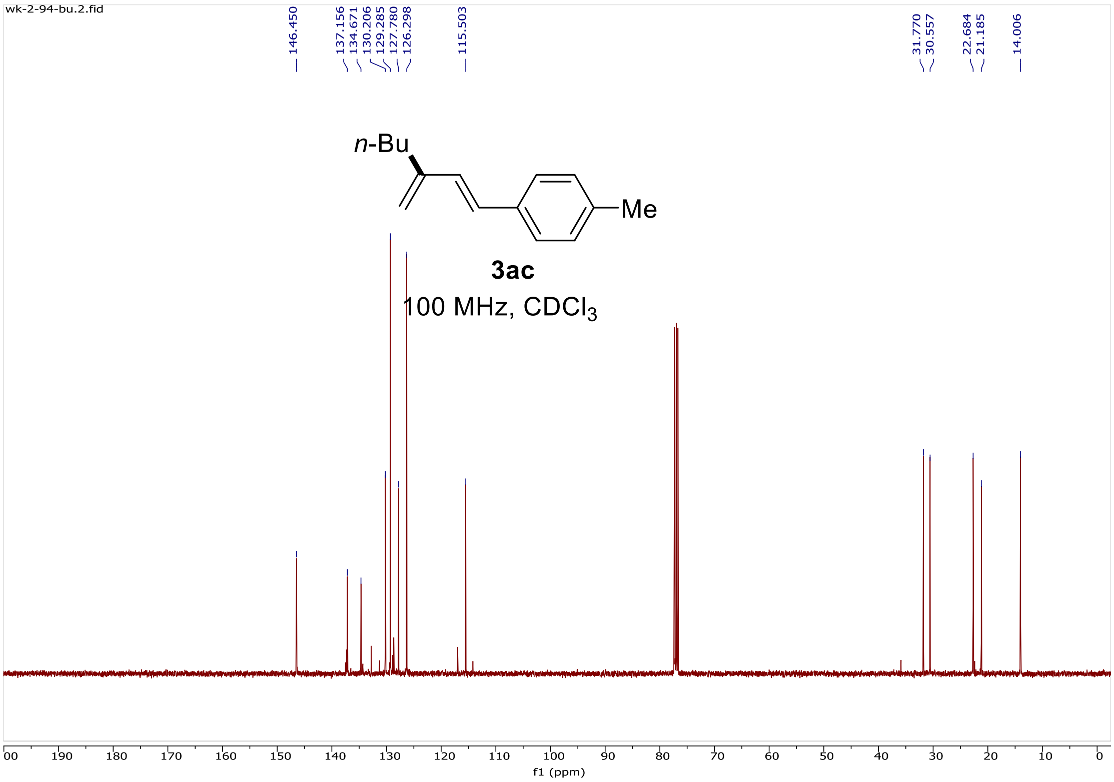
wk-2-94-bu.2.fid
146.450
137.156
134.671
130.206
129.285
127.760
126.298
115.503
31.770
30.557
22.684
21.185
14.006
n-Bu
Me
3ac
100 MHz, CDCl3
f1 (ppm)

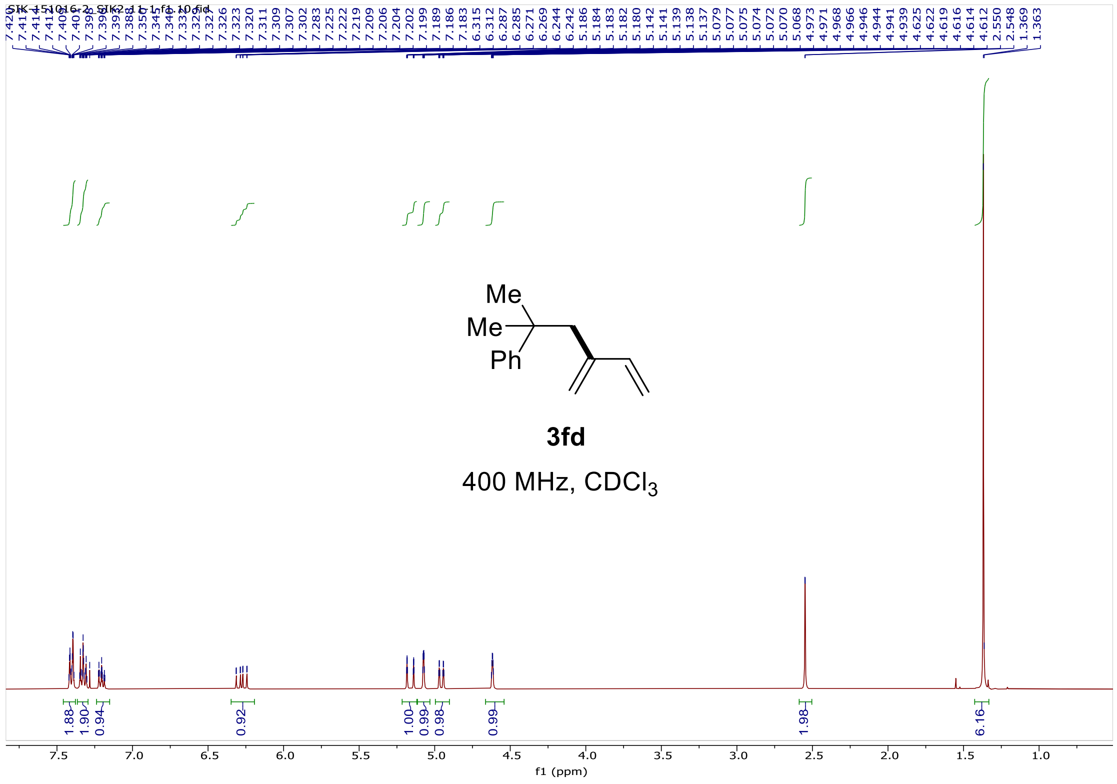
SIK-151016-2_SIK2-11-1-f1.10.fid
Me
Me
Ph
3fd
400 MHz, CDCl3
f1 (ppm)

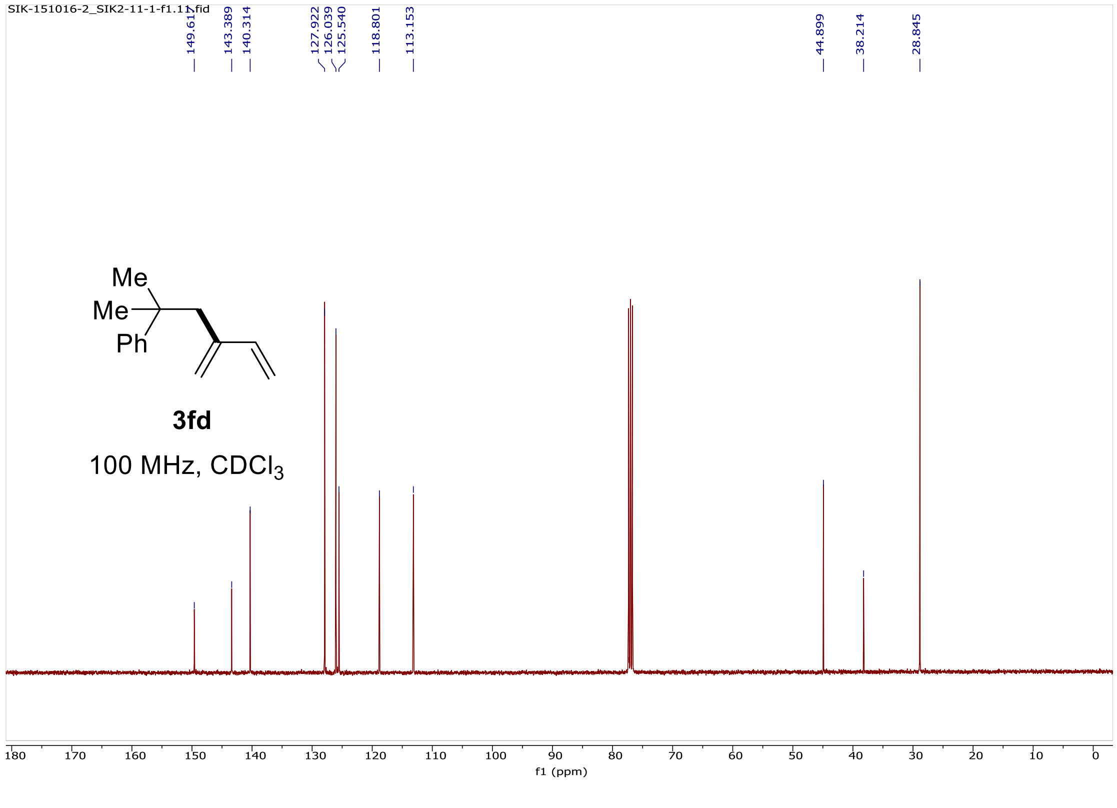
SIK-151016-2_SIK2-11-1-f1.11.fid
149.617
143.389
140.314
127.922
126.039
125.540
118.801
113.153
44.899
38.214
28.845
Me
Me
Ph
3fd
100 MHz, CDCl3
f1 (ppm)

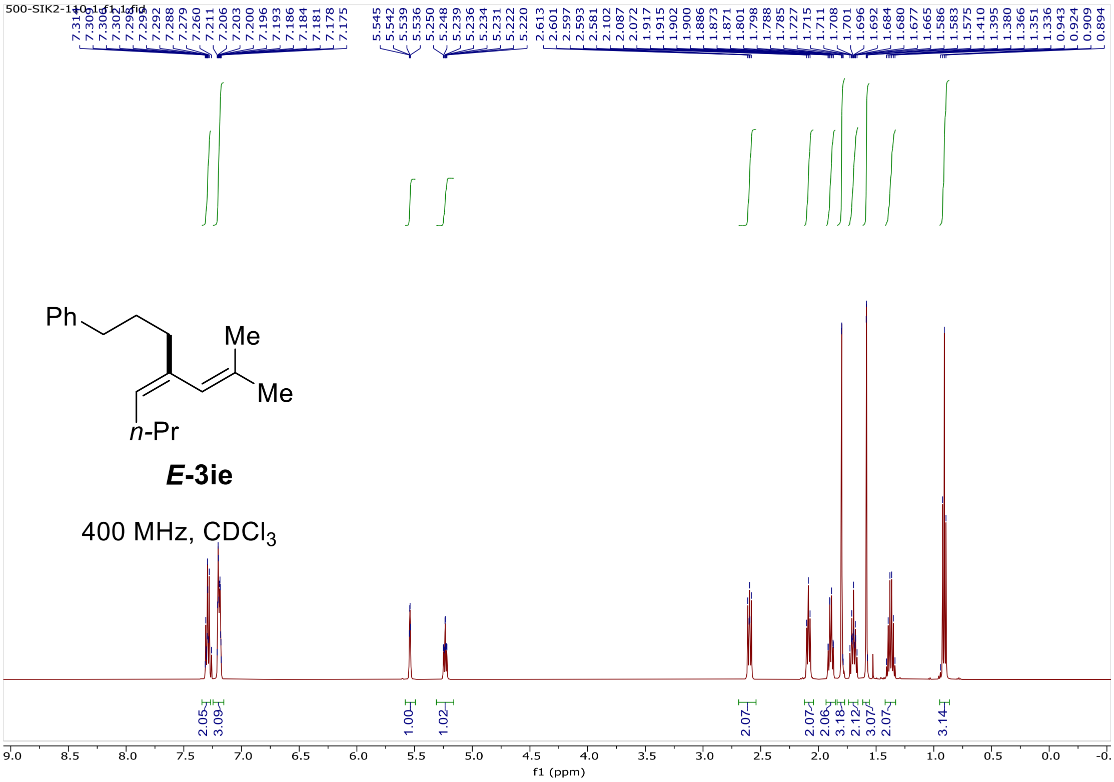
500-SIK2-110-1-f1.1.fid
Ph
Me
Me
n-Pr
E-3ie
400 MHz, CDCl3
2.05
3.09
1.00
1.02
2.07
2.07
2.06
3.18
2.12
3.07
2.07
3.14
f1 (ppm)

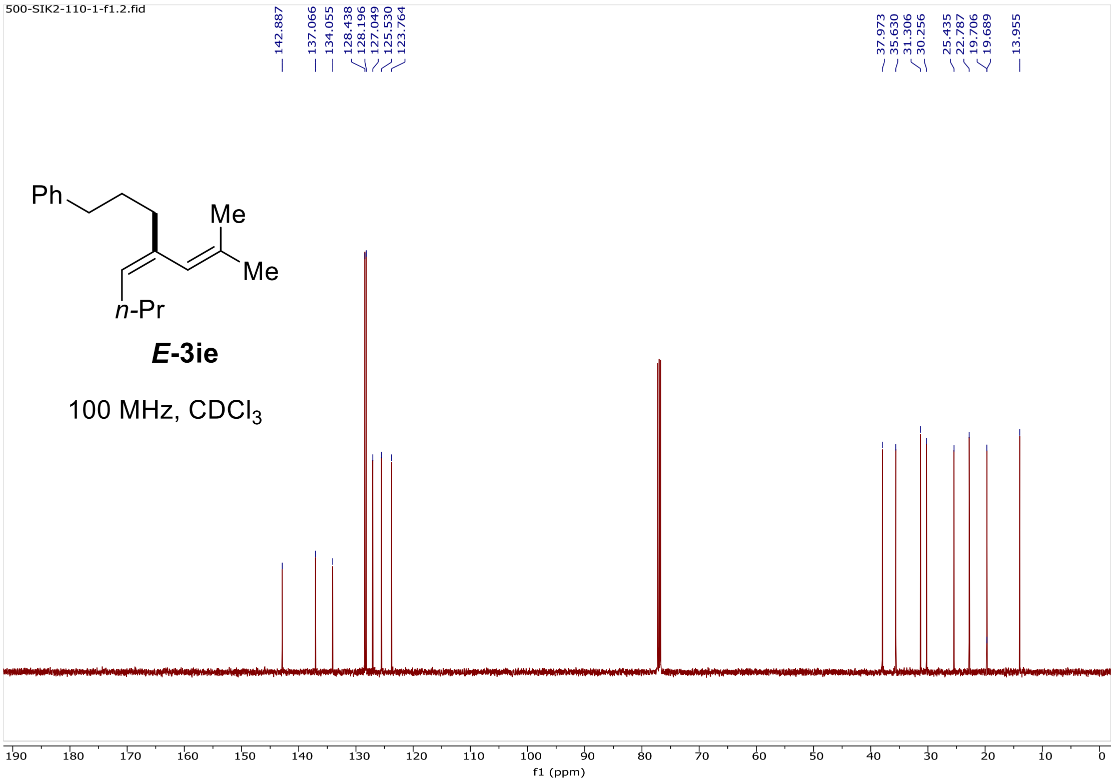
500-SIK2-110-1-f1.2.fid
142.887
137.066
134.055
128.438
128.196
127.049
125.530
123.764
37.973
35.630
31.306
30.256
25.435
22.787
19.706
19.689
13.955
Ph
Me
Me
n-Pr
E-3ie
100 MHz, CDCl3
f1 (ppm)

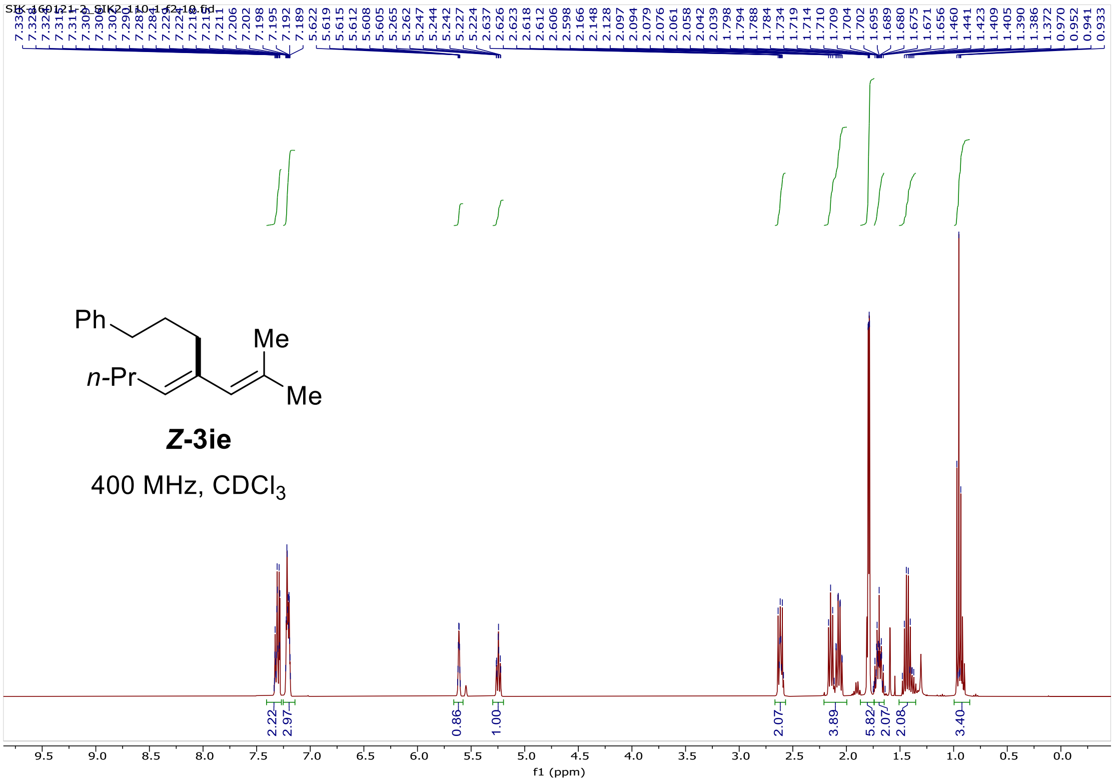
SIK-160121-2_SIK2-110-1-f2.10.fid
Ph
Me
n-Pr
Me
Z-3ie
400 MHz, CDCl3
2.22
2.97
0.86
1.00
2.07
3.89
5.82
2.07
2.08
3.40
f1 (ppm)

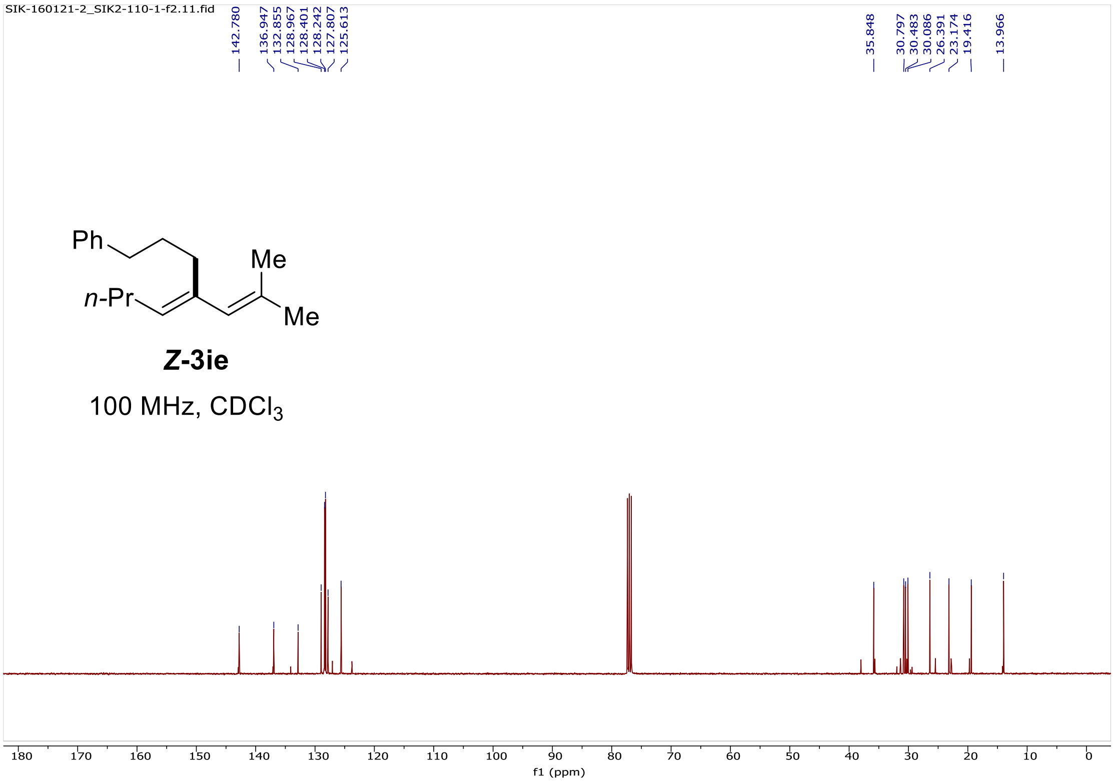
SIK-160121-2_SIK2-110-1-f2.11.fid
142.780
136.947
132.855
128.967
128.401
128.242
127.807
125.613
35.848
30.797
30.483
30.086
26.391
23.174
19.416
13.966
Ph
Me
n-Pr
Me
Z-3ie
100 MHz, CDCl3
f1 (ppm)

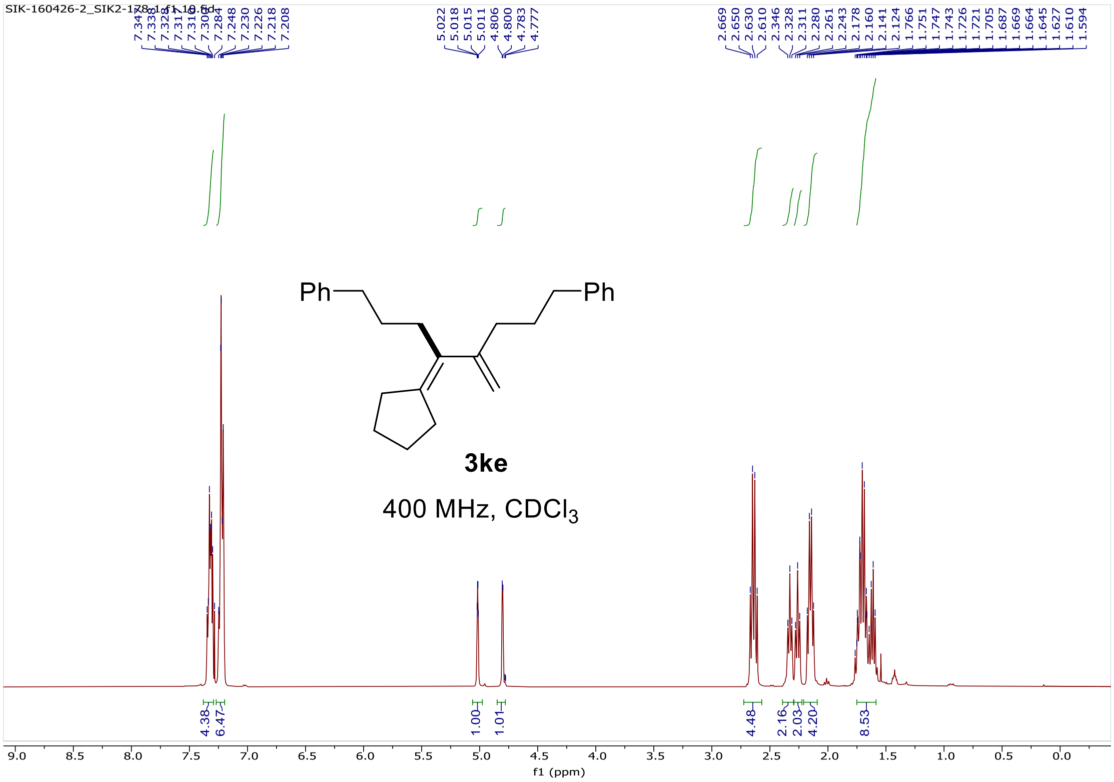
SIK-160426-2_SIK2-178-1-f1.10.fid
Ph
Ph
3ke
400 MHz, CDCl3
f1 (ppm)

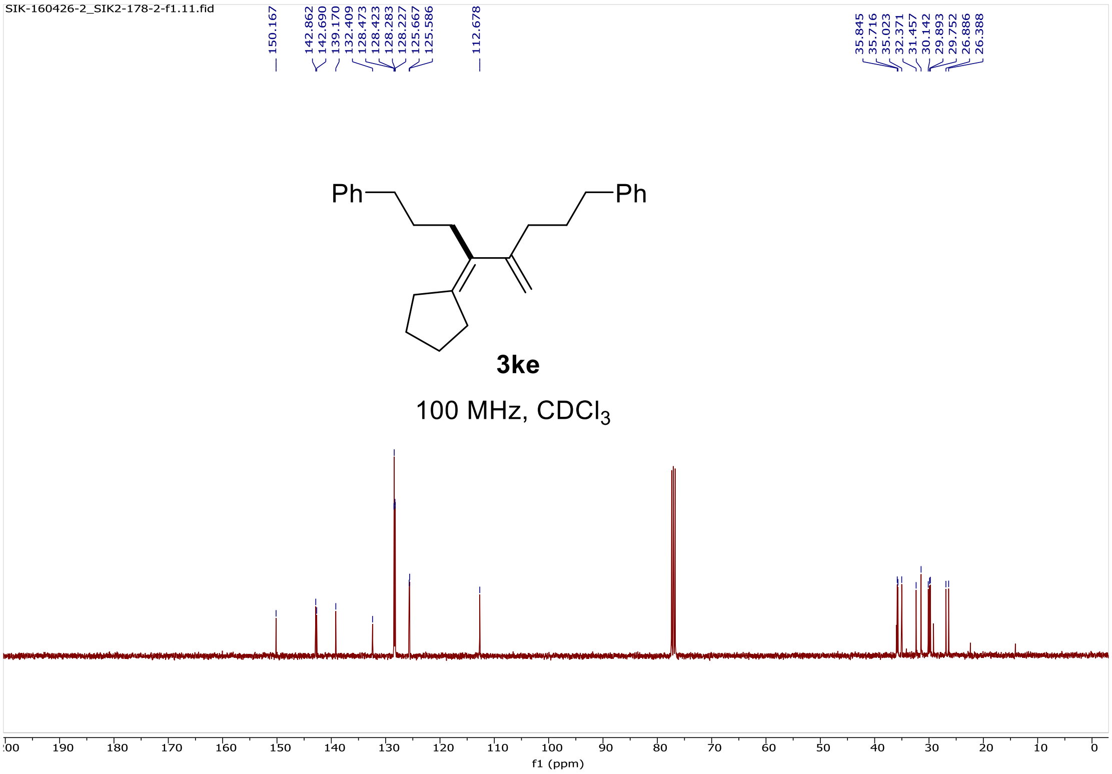
SIK-160426-2_SIK2-178-2-f1.11.fid
150.167
142.862
142.690
139.170
132.409
128.473
128.423
128.283
128.227
125.667
125.586
112.678
35.845
35.716
35.023
32.371
31.457
30.142
29.893
29.752
26.886
26.388
Ph
Ph
3ke
100 MHz, CDCl3
f1 (ppm)

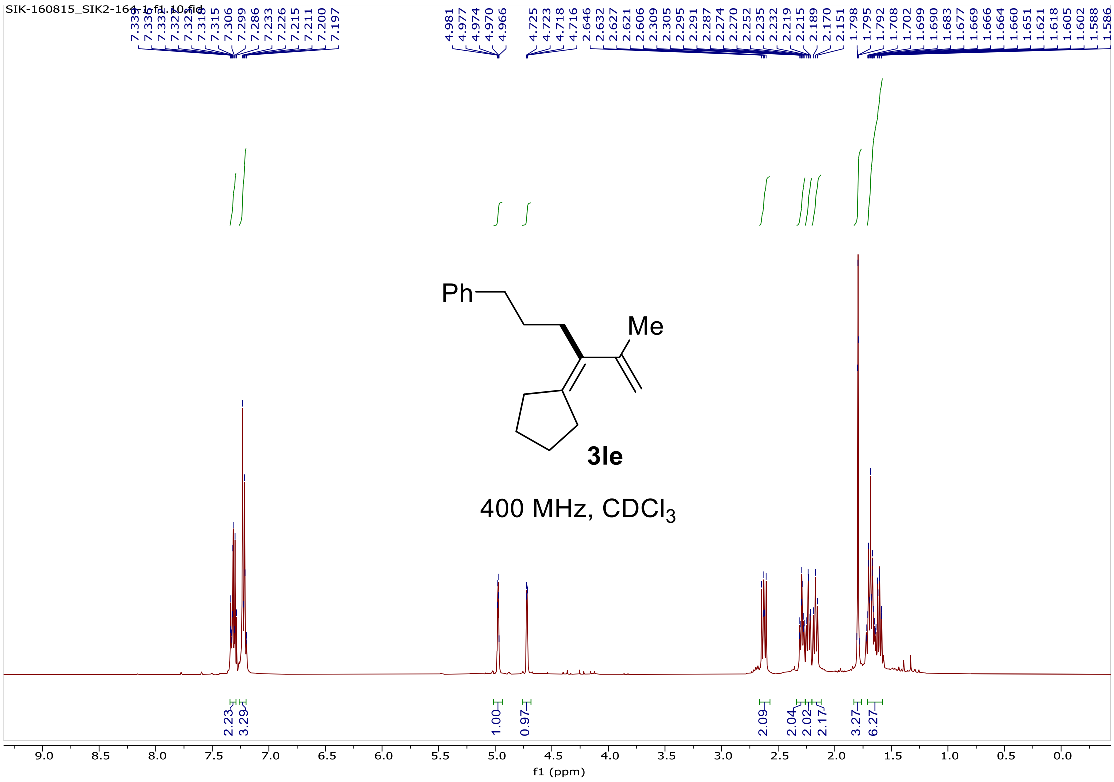
SIK-160815_SIK2-164-1-f1.10.fid
Ph
Me
3le
400 MHz, CDCl3
f1 (ppm)

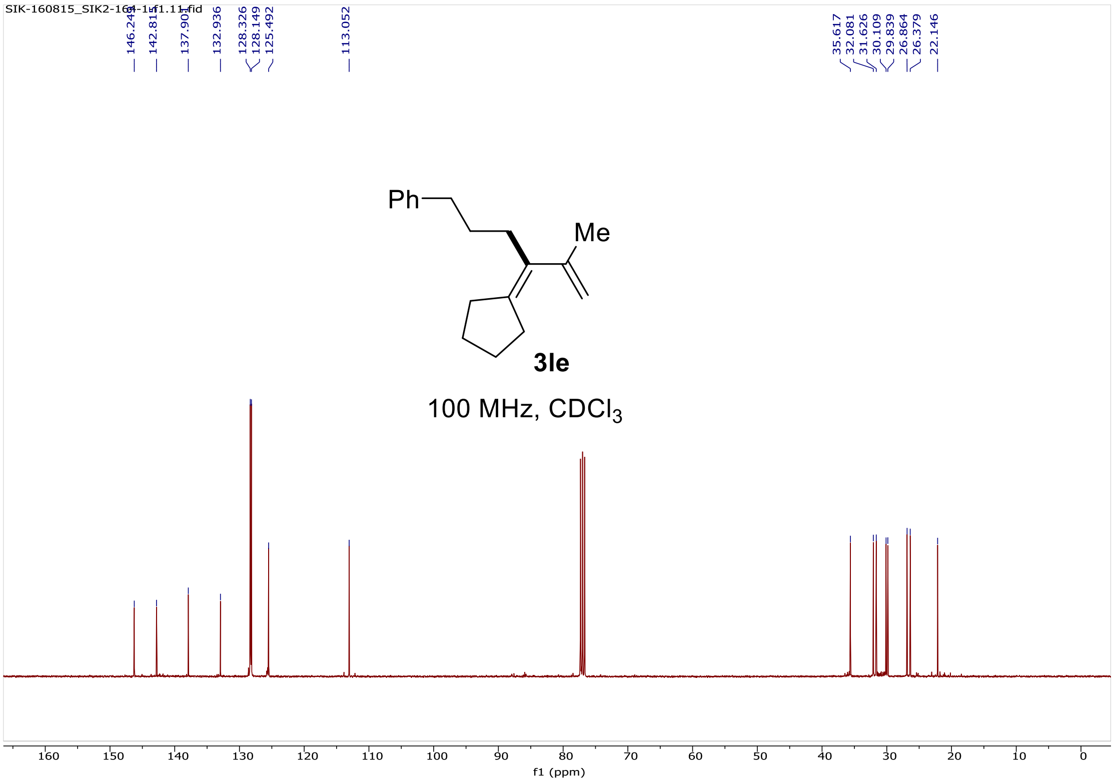
SIK-160815_SIK2-164-1-f1.11.fid
146.24
142.81
137.90
132.936
128.326
128.149
125.492
113.052
35.617
32.081
31.626
30.109
29.839
26.864
26.379
22.146
Ph
Me
3le
100 MHz, CDCl3
f1 (ppm)
